# Pericytes modulate endothelial inflammatory response during bacterial infection

**DOI:** 10.1128/mbio.03252-23

**Published:** 2024-01-30

**Authors:** Thaynara P. Carvalho, Frank A. O. Toledo, Diego F. A. Bautista, Monique F. Silva, Jefferson B. S. Oliveira, Pâmela A. Lima, Fabíola B. Costa, Noelly Q. Ribeiro, Jee-Yon Lee, Alexander Birbrair, Tatiane A. Paixão, Reneé M. Tsolis, Renato L. Santos

**Affiliations:** 1 Departamento de Clínica e Cirurgia Veterinárias, Escola de Veterinária, Universidade Federal de Minas Gerais, Belo Horizonte, Minas Gerais, Brazil; 2 Department of Medical Microbiology and Immunology, University of California, Davis, California, USA; 3 Departamento de Patologia Geral, Instituto de Ciências Biológicas, Universidade Federal de Minas Gerais, Belo Horizonte, Minas Gerais, Brazil; 4 Department of Dermatology, School of Medicine and Public Health, University of Wisconsin-Madison, Madison, Wisconsin, USA; University of Wisconsin-Madison, Madison, Wisconsin, USA

**Keywords:** inflammation, pericytes, endothelial cells, connexin 43, *Brucella*, *Listeria*, *Citrobacter*

## Abstract

Pericytes are located around blood vessels, in close contact with endothelial cells. We discovered that pericytes dampen pro-inflammatory endothelial cell responses. Endothelial cells co-cultured with pericytes had markedly reduced expression of adhesion molecules (PECAM-1 and ICAM-1) and proinflammatory cytokines (CCL-2 and IL-6) in response to bacterial stimuli (*Brucella ovis*, *Listeria monocytogenes*, or *Escherichia coli* lipopolysaccharide). Pericyte-depleted mice intraperitoneally inoculated with either *B. ovis*, a stealthy pathogen that does not trigger detectable inflammation, or *Listeria monocytogenes*, developed peritonitis. Further, during *Citrobacter rodentium* infection, pericyte-depleted mice developed severe intestinal inflammation, which was not evident in control mice. The anti-inflammatory effect of pericytes required connexin 43, as either chemical inhibition or silencing of connexin 43 abrogated pericyte-mediated suppression of endothelial inflammatory responses. Our results define a mechanism by which pericytes modulate inflammation during infection, which shifts our understanding of pericyte biology: from a structural cell to a pro-active player in modulating inflammation.

**IMPORTANCE:**

A previously unknown mechanism by which pericytes modulate inflammation was discovered. The absence of pericytes or blocking interaction between pericytes and endothelium through connexin 43 results in stronger inflammation, which shifts our understanding of pericyte biology, from a structural cell to a player in controlling inflammation.

## INTRODUCTION

Pericytes comprise a heterogeneous population of cells that are located around blood vessels in the perivascular space, in close contact with endothelial cells. Pericytes retain the capacity for proliferation and differentiation so they may be considered to be mesenchymal stem cells (1–3). In the vessel wall, pericytes are in close contact with endothelial cells, which during inflammation express adhesion molecules to promote transendothelial migration of neutrophils toward sites of inflammation (4). Pericytes are also involved in this process by directly interacting with neutrophils through adhesion molecules, driving the neutrophils through gaps that facilitate neutrophil migration (5). However, emerging evidence demonstrating the involvement of pericytes in many pathological conditions points to additional functions of pericytes that are yet to be discovered (6).

Pericytes and endothelial cells have physical and functional interactions through peg-socket junctions, which are considered essential for the stability of blood vessels (1, 7). Peg-socket junctions anchor these two cell types together, allowing exchanges between them through connexins, particularly connexin 43 (Cx43) (8, 9). Cx43 channels allow the transfer of ions, second messengers such as cAMP, and other small molecules between pericytes and endothelial cells (10–12).

The role of pericytes in angiogenesis and control of blood flow is well known (13–15). Additionally, pericytes play a role in innate immune responses. During acute inflammation induced by tumor necrosis factor alpha (TNF-α) or lipopolysaccharide (LPS), post capillary pericytes express C-X-C Ligand 1 (CXCL1), which drives transmigration of neutrophils to the site of tissue damage (16). Furthermore, activated precapillary and capillary pericytes express intercellular adhesion molecule 1 (ICAM-1), macrophage migration inhibitory factor (MIF), C-C motif chemokine ligand 2 (CCL-2), and C-X-C motif chemokine ligand 8 (CXCL-8) in order to attract and activate transmigrated neutrophils and macrophages (17). However, pericytes also have immunosuppressive properties: when these cells are co-cultured with activated T cells, there is an impairment of T cell proliferation and production of interferon gamma (IFN-γ) and TNF-α (18). In the retina, loss of pericytes during early stages of diabetic retinopathy results in increased leukocyte influx, hemorrhage, and microvascular lesions (18, 19), which is associated with increased production of CCL-2, interleukin-6 (IL-6), and TNF-α (19). Pericytes also contribute to immunosuppression in the glioblastoma microenvironment *in vitro*, where pericytes negatively correlated with leukocyte recruitment and influx of CD8^+^ T cells (20).

During bacterial infection, invading pathogens are recognized by the host innate immune system that senses microbe-derived molecules known as pathogen-associated molecular patterns (PAMPs), which include LPS, peptidoglycans, lipoproteins, adhesins, enzymes, toxins, and nucleic acids. These molecules are recognized by Toll-like receptors (TLRs) (21). Once PAMPs are sensed, innate immune cells trigger a pro-inflammatory response (22). However, some pathogens can escape detection by innate immunity. For instance, LPS of *Brucella* spp., which is a facultative intracellular Gram-negative bacterial pathogen, has a non-canonical lipid A, making it a very weak TLR4 agonist (23–27). This feature of *Brucella* spp. makes them excellent tools to interrogate innate immune responses that are not dominated by TLR signaling.

The role of pericytes during bacterial infections in general remains largely unknown. Based on the role of pericytes in inducing and suppressing inflammation in the context of different pathologic conditions, we aimed to test the hypothesis that pericytes modulate inflammation during bacterial infections.

## RESULTS

### Pericyte cells are not permissive to *Brucella ovis* infection

**Fig 1:**
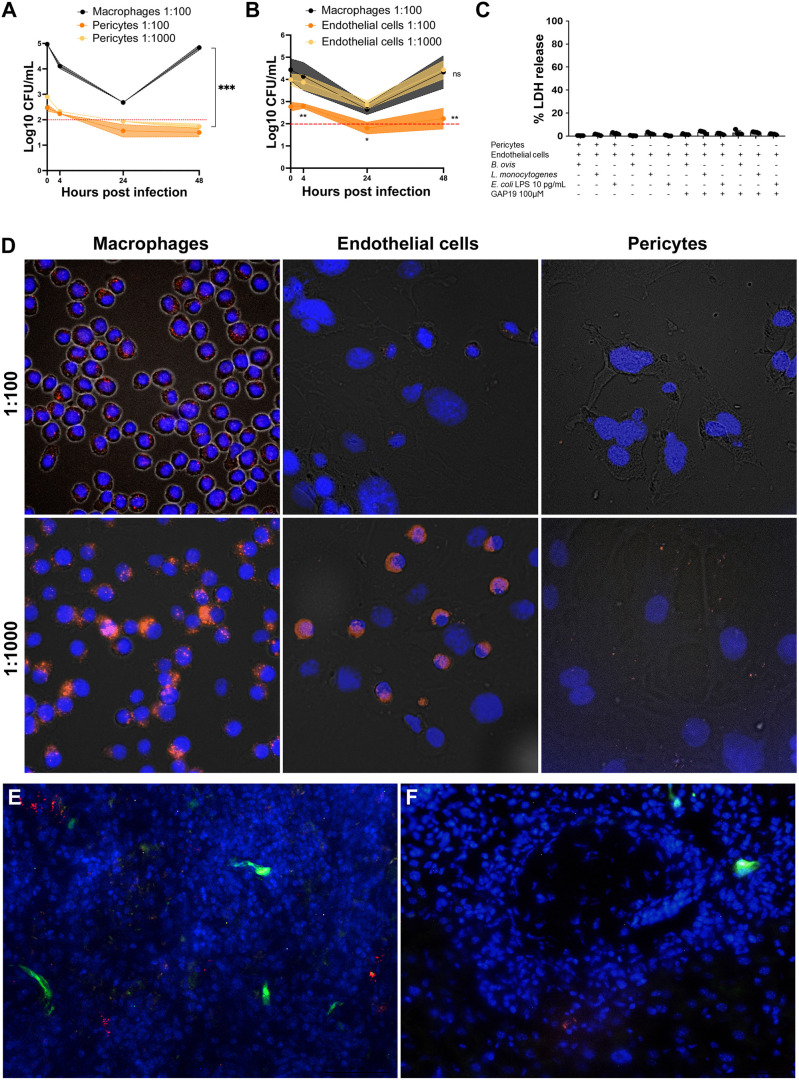
Pericytes are not permissive to *Brucella ovis* infection. (**A**) *B. ovis* invasion and intracellular survival/multiplication in macrophages (MOI 1:100) or pericytes (MOI 1:100 or 1:1,000); the red dashed line indicates the detection limit. (**B**). *B. ovis* invasion and intracellular survival/multiplication in macrophages (MOI 1:100) or endothelial cells (MOI 1:100 or 1:1,000); the red dashed line indicates the detection limit. (**C**) Lactate dehydrogenase (LDH) release assay as indicator of cytotoxicity under various experimental conditions performed in this study. (**D**) Macrophages, endothelial cells, or pericytes were inoculated with mCherry-expressing *B. ovis* (MOI 1:100 or 1:1,000) under conditions similar to those in experiments described in panels **A** and **B**. (**E, F**) Nestin^+^ pericytes constitutively expressing GFP (green fluorescent protein) did not colocalize with mCherry-expressing *B. ovis* (red) in the liver (**E**) and spleen (**F**) of mice experimentally infected at 6 days post infection (dpi). Data are representative of three independent experiments performed in triplicates. *P < 0.05, **P < 0.01, and ***P < 0.001; ns: non-significant (*P* > 0.05).

In order to assess the influence of pericytes on endothelial-mediated inflammation, we elected to use a microbe that does not trigger strong inflammatory response. *Brucella ovis* was selected as a model organism to assess the role of pericytes in modulating inflammation due to its low intrinsic pro-inflammatory potential. Considering that *Brucella* can invade, survive, and multiply in many different cells *in vitro* and *in vivo* (28), we initially evaluated whether pericytes can serve as a niche for *B. ovis* infection. To this end, we inoculated cultured pericytes, endothelial cells, and macrophages with *B. ovis*. As expected, based on previous studies (29), *B. ovis* was able to invade and survive in macrophages, with an initial decrease in intracellular colony forming unit (CFU) numbers over the first 24 hours post infection (hpi), followed by intracellular multiplication from 24 to 48 hpi (Fig. 1A and B). Similarly, *B. ovis* invaded, survived, and multiplied in endothelial cells with kinetics similar to those observed in macrophages (Fig. 1B). However, a multiplicity of infection (MOI) of 1,000 was required to achieve intracellular CFU numbers that were similar to those measured in macrophages infected with a MOI of 100. These results indicated that endothelial cells are permissive to *Brucella* spp. infection and multiplication. In contrast, *B. ovis* invaded pericytes in significantly lower numbers regardless of the MOI (either 100 or 1,000). Importantly, there was no intracellular multiplication of *B. ovis* in pericytes with intracellular numbers remaining close to the limit of detection at 24 or 48 hpi (Fig. 1A). Therefore, our data indicated that pericytes are less permissive than endothelial cells or macrophages to the internalization of *B. ovis* and are not permissive to its intracellular multiplication.

*B. ovis* is known to have a low cytotoxicity for most cells (30), but as the results can be influenced by the host cell death itself due to the extracellular exposure to gentamycin used in the experiments, we decided here to use the LDH release as an indicator of host cell death after *B. ovis* infection. Therefore, LDH concentrations were measured in the supernatant of cultured pericytes, endothelial cells, and macrophages at 24 hpi with *B. ovis* (MOI 100). As predicted, cell death in *B. ovis*-inoculated pericytes, macrophages, and endothelial cells was very low as demonstrated by a minimum LDH release (Fig. 1C), indicating that intracellular CFU counts were not affected by host cell death in this study.

Next, we evaluated the distribution of *B. ovis* in inoculated cultured cells. In good agreement with the intracellular CFU numbers, fluorescence microscopy demonstrated that *B. ovis* infected the majority of macrophages and endothelial cells in culture but only occasionally infected pericytes (Fig. 1D).

In order to evaluate the interaction or tropism of *B. ovis* to pericytes *in vivo*, we infected nestin-GFP mice, which have nestin^+^ pericytes that constitutively express GFP, with *B. ovis-*mCherry (10^6^ CFU/mice), and sections of the liver and spleen were analyzed by fluorescent microscopy at 6 dpi. Fluorescent *B. ovis*-mCherry was detected as clusters associated with host cells, but it did not colocalize with pericytes in any of the samples evaluated (Fig. 1E and F).

Together, these results support the notion that pericytes are less permissive to *B. ovis* invasion and intracellular multiplication, when compared with endothelial cells and macrophages. Furthermore, there is no evidence of *B. ovis* tropism to pericytes *in vivo* in the mouse.

### Pericytes downregulate expression of inflammatory mediators and adhesion molecules in endothelial cells stimulated with *B. ovis*

**Fig 2:**
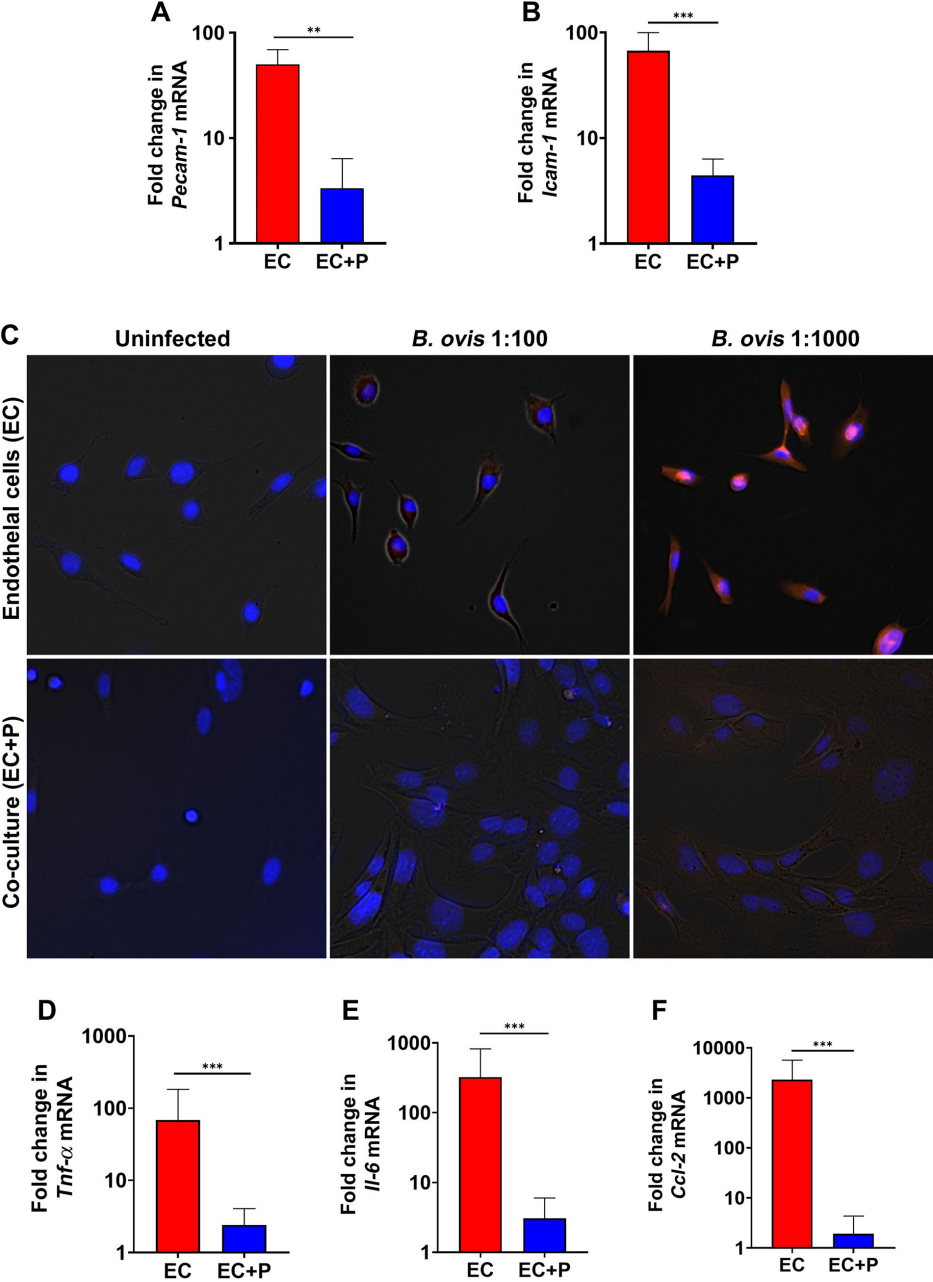
Pericytes downregulate expression of adhesion molecules and inflammatory mediators by endothelial cells inoculated with *Brucella ovis*. (**A, B**) Relative transcription levels (fold change in comparison to uninfected) of *Pecam-1* (**A**) or *Icam-1* (**B**) by endothelial cells (EC) or endothelial cells co-cultured with pericytes (EC + P) inoculated with *B. ovis*. (**C**) Expression of PECAM-1 (red) by endothelial cells or endothelial cells co-cultured with pericytes, uninfected or infected with *B. ovis* (MOI 1:100 or 1:1,000). (**D–F**) Relative transcription levels (fold change in comparison to uninfected) of *Tnf-α* (**D**), *Il-6* (**E**), or *Ccl-2* (**F**) by EC or EC + P inoculated with *B. ovis*. Data in panels A, B, D, E, and F are representative of three independent experiments performed in triplicate. Data in panel C are from a single experiment, performed in triplicate. **P < 0.01 and ***P < 0.001.

Considering the role of pericytes in controlling leukocyte migration through the vascular wall (19, 31) and the expression of immunoglobulin (Ig)-like cell adhesion molecules (CAMs), namely, ICAM-1/CD54, ICAM-2/CD102, VCAM-1/CD106, and PECAM-1/CD31 (32, 33) by endothelial cells, which is a key step during the initial phase of acute inflammation, we assessed whether pericytes influence expression of adhesion molecules by endothelial cells during infection with *B. ovis* (MOI 100). Transcriptional levels of *Pecam-1* (also known as CD31) and *Icam-1* (also known as CD54) were determined at 24 hpi. When endothelial cells were co-cultured with pericytes, there was downregulation of *Pecam-1* and *Icam-1* when compared with endothelial cells alone with reductions to 0.8% and 2.6% of the mRNA levels of *Pecam-1* and *Icam-1* at 24 hpi, respectively (Fig. 2A and B). These results suggest that pericytes downregulate transcription levels of *Pecam-1* and *Icam-1* in endothelial cells. To further characterize reduction of adhesion molecules when endothelial cells are in contact with pericytes, we performed immunofluorescent detection of PECAM-1. Endothelial cells alone had marked expression of PECAM-1 when inoculated with *B. ovis* (Fig. 2C), whereas expression of PECAM-1 was markedly suppressed when endothelial cells were co-cultured with pericytes (Fig. 2C).

In addition to expression of adhesion molecules, endothelial cells enhance the inflammatory response by triggering expression of proinflammatory genes such as *Tnf-α*, *Il-6*, and *Ccl-2* when exposed to PAMPs (34, 35). Therefore, transcription levels of *Tnf-α*, *Il-6*, and *Ccl-2* were measured in endothelial cells alone or endothelial cells co-cultured with pericytes at 24 hpi with *B. ovis. Tnf-α*, *Il-6*, and *Ccl-2* were upregulated in endothelial cells infected with *B. ovis* with increases of 437.84-, 2,365.82-, and 343.39-fold in mRNA levels at 24 hpi, respectively (Fig. 2D, E, and F). Interestingly, transcription of these proinflammatory mediators was markedly reduced when endothelial cells were co-cultured with pericytes at 24 hpi with *B. ovis*. These results indicate that pericytes suppress expression of adhesion molecules and inflammatory mediators by cultured endothelial cells during the course of *B. ovis* infection.

### Pericyte modulation of endothelial inflammatory response is conserved in response to various pathogens

**Fig 3:**
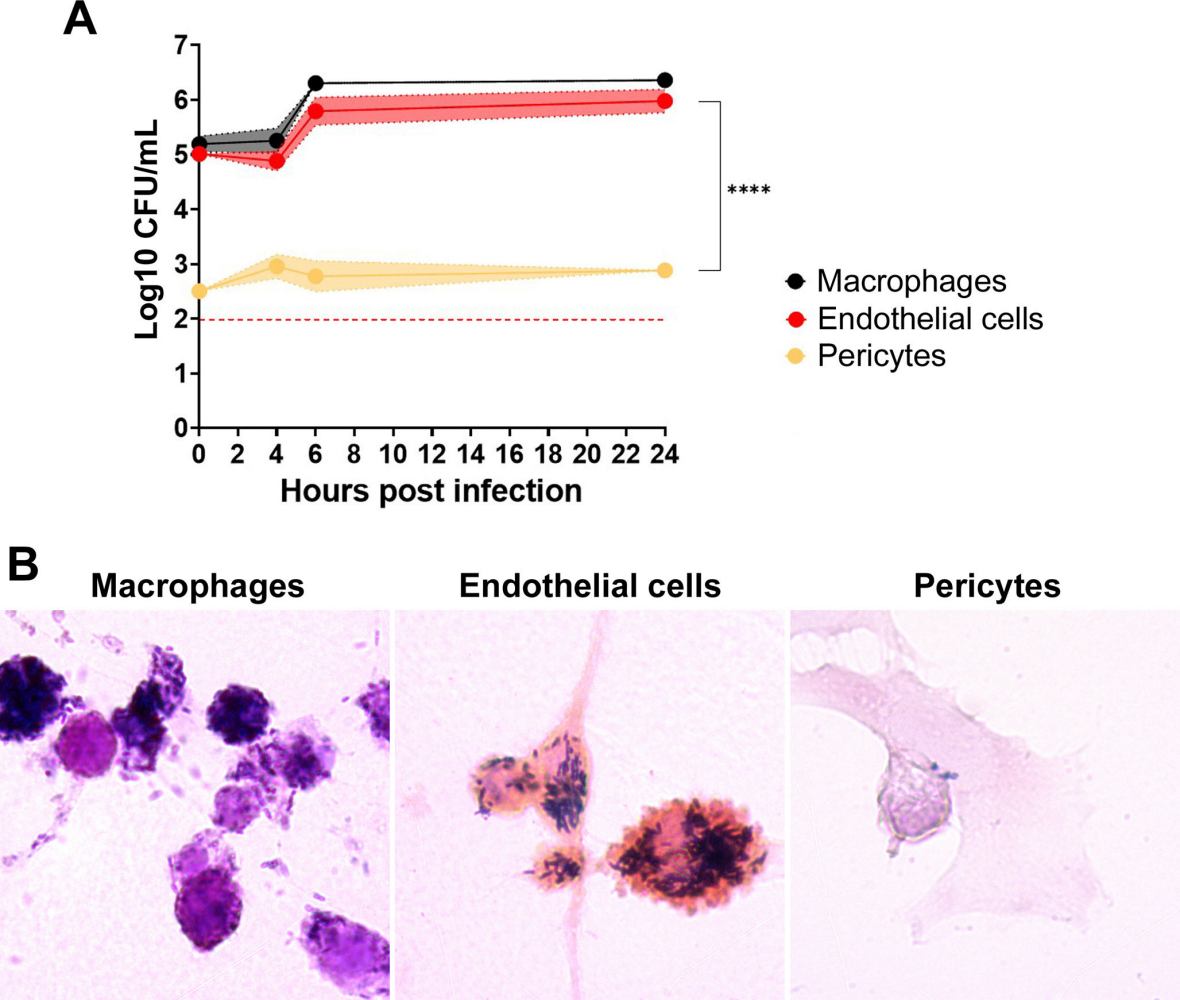
Pericytes are not permissive to *Listeria monocytogenes* infection. (**A**) Internalization, survival, and multiplication of *Listeria monocytogenes* in macrophages, endothelial cells, and pericytes (****P < 0.0001). (**B**) Intracellular *L. monocytogenes* stained in blue (Gram positive) in macrophages, endothelial cells, and pericytes experimentally inoculated. Data are representative of three independent experiments performed in triplicate.

In order to evaluate whether the modulation of expression of adhesion molecules and inflammatory mediators was specific to *B. ovis*, different stimuli were employed: *Listeria monocytogenes* (MOI 5) or purified *Escherichia coli* LPS (10 pg/mL). Considering the possibility of cytotoxicity, LDH was measured in the supernatant of cultures exposed to these stimuli. Endothelial cells cultured alone or co-cultured with pericytes were stimulated with *E. coli* LPS or inoculated with *L. monocytogenes.* Both treatments elicited minimal LDH release (~less than 10%) (Fig. 1C). Similar to results with *B. ovis*, pericytes were not permissive to infection or intracellular growth of *L. monocytogenes* (Fig. 3A and B).

**Fig 4:**
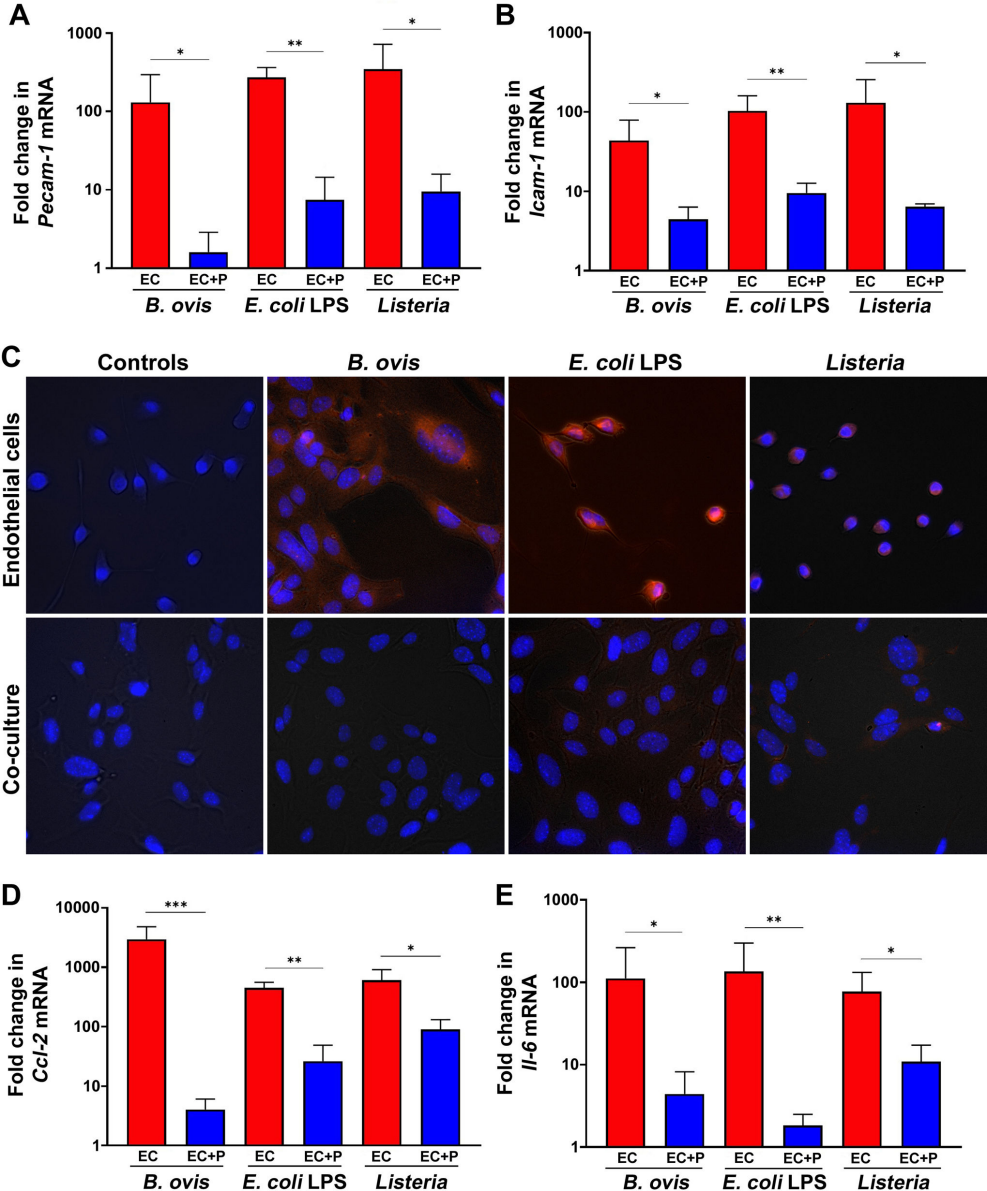
Pericyte modulation of endothelial inflammatory response to *Brucella ovis*, *Listeria monocytogenes*, or *Escherichia coli* LPS. (**A, B**) Relative transcription levels (fold change in comparison to uninfected) of *Pecam-1* (**A**) or *Icam-1* (**B**) by endothelial cells (red columns) or endothelial cells co-cultured with pericytes (blue columns) inoculated with *B. ovis* and *L. monocytogenes* or stimulated with *E. coli* LPS. (**C**) Expression of PECAM-1 (red) by endothelial cells or endothelial cells co-cultured with pericytes, uninfected or inoculated with *B. ovis*, *E. coli* LPS, or *L. monocytogenes*. Images of PECAM-1 expression in cells cultured with *B. ovis* are from the same experiment shown in Fig. 2C and are shown here again for comparison. (**D, E**) Relative transcription levels (fold change in comparison to uninfected) of *Ccl-2* (**D**) or *Il-6* (**E**) by endothelial cells (red columns) or endothelial cells co-cultured with pericytes (blue columns) inoculated with *B. ovis* and *L. monocytogenes* or stimulated with *E. coli* LPS. Data are representative of three independent experiments performed in triplicates. *P < 0.05, **P < 0.01, and ***P < 0.001.

Consistent with our previous experiments, *B. ovis* triggered expression of adhesion molecules and inflammatory mediators. Both *L. monocytogenes* and *E. coli* LPS also triggered expression of adhesion molecules (*Pecam-1* and *Icam-1*) (Fig. 4A and B), and PECAM-1 expression was further characterized by immunofluorescence (Fig. 4C). *L. monocytogenes* and *E. coli* LPS induced transcription of pro-inflammatory genes (*Ccl-2* and *Il-6*) as demonstrated by mRNA levels (Fig. 4D and E). In contrast, when endothelial cells were co-cultured with pericytes, there was a marked downregulation of adhesion molecules and inflammatory mediators after inoculation with *L. monocytogenes* or *E. coli* LPS under the same experimental conditions (Fig. 4A through E). These results indicate that the modulation of endothelial cell inflammatory response by pericytes is not specific to *B. ovis* infection but rather occurs in response to various inflammatory stimuli such as a Gram-positive pathogen (*L. monocytogenes*) or a strong TLR4 agonist (*E. coli* LPS).

### The stealthy pathogen *Brucella ovis* induces marked acute inflammation *in vivo* in pericyte-depleted mice

**Fig 5:**
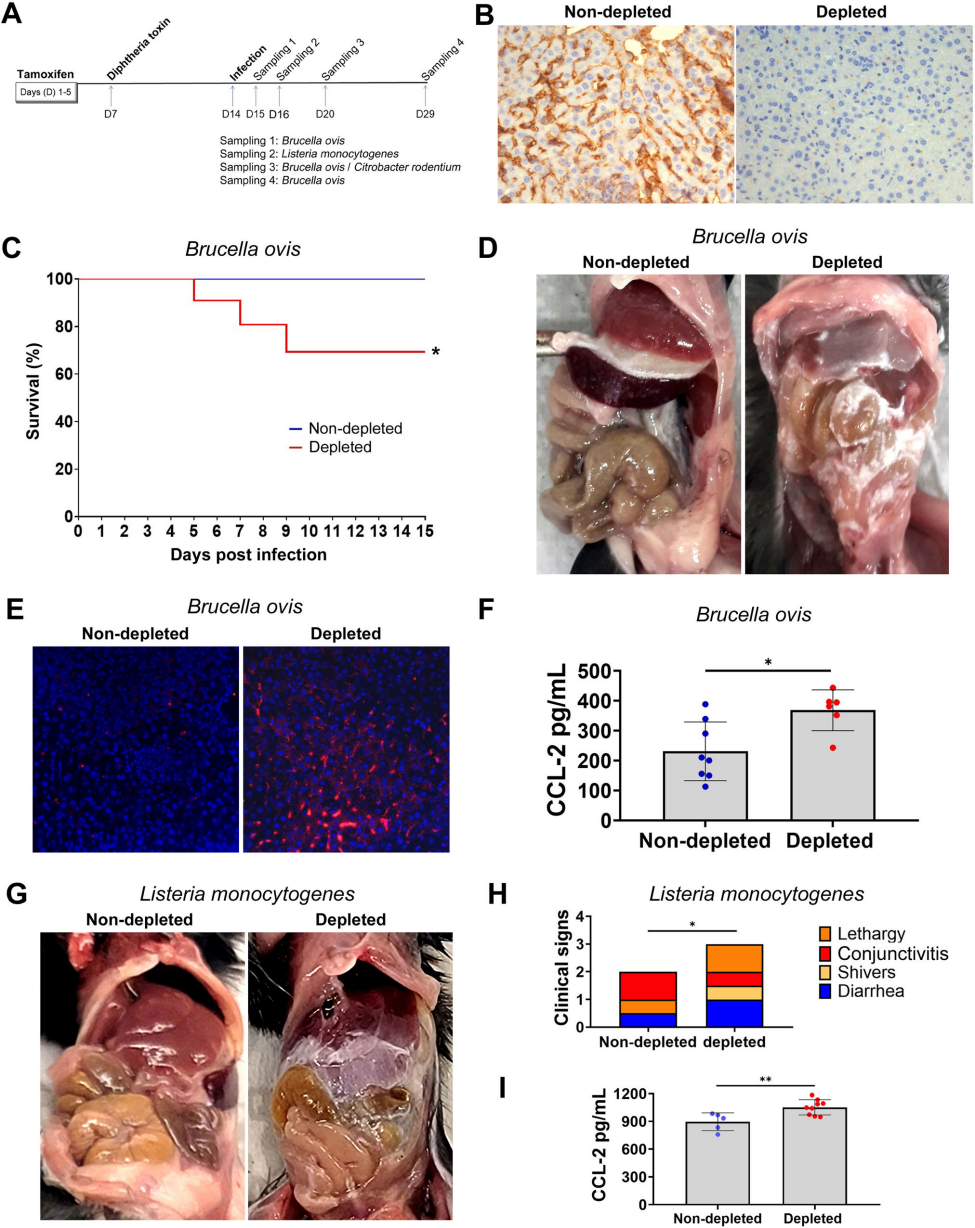
Pericytes modulate the inflammatory response to bacterial pathogens *in vivo*. (**A**) Time line for depletion of pericytes (administration of tamoxifen followed by diphtheria toxin [DT]), experimental infection, and sampling (S). (**B**) Confirmation of depletion of NG2 + pericytes by immunohistochemistry in the liver. (**C**) Survival curve of pericyte-depleted (*n* = 10) or non-depleted (*n* = 14) mice infected with *Brucella ovis* (*P < 0.05). (**D**) Abdominal cavity of non-depleted and pericyte-depleted mice infected with *B. ovis*, which is associated with a diffuse fibrinous peritonitis in pericyte-depleted mice at 6 dpi. (**E**) Detection of *Pecam-1* mRNA (red) by *in situ* hybridization in the liver of non-depleted or pericyte-depleted mice infected with *Brucella ovis*. (**F**) Serum levels of CCL-2 in non-depleted (*n* = 10) and pericyte-depleted (*n* = 6) mice infected with *B. ovis* (*P < 0.05). (**G**) Abdominal cavity of non-depleted and pericyte-depleted mice infected with *Listeria monocytogenes*, which is associated with a diffuse fibrinous peritonitis in pericyte-depleted mice at 2 dpi. (**H**) Clinical sign score of non-depleted (*n* = 4) and pericyte-depleted (*n* = 7) mice infected with *Listeria monocytogenes* (*P < 0.05). (**I**) Serum levels of CCL-2 in non-depleted (*n* = 4) and pericyte-depleted (*n* = 7) mice infected with *L. monocytogenes* (*P < 0.05).

The neuron-glial antigen 2 proteoglycan (NG2) is one of the most reliable known markers for arteriolar and capillary pericytes (36, 37). The NG2 glycoprotein, also known as chondroitin sulfate proteoglycan-4 (CSPG-4), is a cell surface component that plays an essential role in pericyte maturation, inducing proliferation and motility, favoring tissue remodeling and neovascularization (38–42). To investigate the role of pericytes in modulating acute inflammation *in vivo*, we used double-transgenic mice (NG2creER × iDTR), in which NG2-expressing pericytes can be induced to express diphtheria toxin receptor (DTR) after treatment with tamoxifen. This allows for selective depletion of NG2-expressing pericytes upon treatment of transgenic mice with tamoxifen followed by diphtheria toxin. Isogenic iDTR C57BL/6 mice (without depletion of pericytes but subjected to the same treatment protocol) were used as controls. Mice were infected intraperitoneally (IP) with 10^6^ CFU of *B. ovis* and were sampled at 1, 6, and 15 dpi (Fig. 5A). Depletion of NG2^+^ pericytes was confirmed by immunohistochemistry using a monoclonal antibody targeting the NG2 glycoprotein on liver samples (Fig. 5B).

We next determined the effect of pericyte depletion on *B. ovis* infection. Based on previous studies, we did not expect *B. ovis* to cause lethal infections in mice (29), which was confirmed in our control mice that exhibited no clinical signs of infection. In contrast, 5 out of 10 pericyte-depleted mice developed clinical signs starting at 4 dpi. These mice had lethargy, curved backs, bristly pelts, shaking, and abdominal rigidity, with unexpected lethal outcomes in 30% (3/10) of mice (Fig. 5C). Infected mice were sampled at 1, 6, and 15 dpi, allowing an assessment of pathologic changes during the course of infection. At 1 dpi, none of the mice had gross changes, but at 6 or 15 dpi, all pericyte-depleted mice infected with *B. ovis* developed acute fibrinous peritonitis. The abdominal cavities of pericyte-depleted mice that were infected with *B. ovis* contained variable amounts of fibrin adhered to the surface of the liver, spleen, and other abdominal organs, characterizing a severe diffuse fibrinous peritonitis (Fig. 5D). In contrast, neither the control (non-depleted) infected mice nor the mock-infected, pericyte-depleted mice developed peritonitis (Fig. 5D). All infected mice (pericyte depleted or non-depleted) developed splenomegaly with multifocal white-yellow areas in the liver and spleen, which microscopically corresponded to microgranulomas containing immunolabeled intralesional *Brucella* sp. (Fig. S1). Although intralesional *B. ovis* was detected in all infected mice, immunostaining was more intense in the liver and spleen from pericyte-depleted mice compared with non-depleted mice (Fig. S1). Microscopic analysis confirmed the diagnosis of fibrinous peritonitis in pericyte-depleted mice infected with *B. ovis*. The hepatic parenchyma adjacent to the capsule had marked hepatocellular degeneration (cytoplasmic vacuolation and nuclear pyknosis), which were not observed in non-depleted and infected mice (Fig. S2). Paralleling what was observed in infected cultured cells, pericyte-depleted mice infected with *B. ovis* had increased transcription of *Pecam-1* in the liver as demonstrated by *in situ* hybridization (Fig. 5E) and higher levels of CCL-2 in the serum when compared with non-depleted infected mice (Fig. 5F).

To ensure that peritonitis in pericyte-depleted mice were not due to other opportunistic pathogens, abdominal contents from pericyte-depleted and infected mice (with peritonitis) as well as from non-depleted and infected mice or uninfected controls were cultured for bacterial isolation. No pathogenic or opportunistic microorganism other than *B. ovis* in the case of infected mice was cultured from the abdominal cavity, demonstrating that the peritonitis in infected pericyte-depleted mice was caused by *B. ovis* infection.

Together, these results indicate that, as demonstrated in cultured cells, pericytes restrain the acute inflammatory response *in vivo* since *B. ovis*, which is a stealthy pathogen that does not trigger inflammation when inoculated intraperitoneally in mice, is capable of inducing an acute severe inflammatory response in the abdominal cavity in the absence of pericytes.

### Live virulent *Brucella ovis* is required for eliciting inflammation in pericyte-depleted mice

Fibrinous peritonitis was a consistent finding in pericyte-depleted mice intraperitoneally infected with *B. ovis. Brucella* sp. and other intracellular bacteria developed strategies for intracellular survival and multiplication. The *virB* operon-encoded type IV secretion system (T4SS) is required for the intracellular survival of *Brucella* spp. (43). Indeed, strains lacking a functional T4SS are not capable of evading lysosomal degradation and neither replicate nor survive in host cells. In this context, we investigated whether a mutant *B. ovis* strain lacking a functional T4SS or inactivated *B. ovis* (gamma irradiated) could induce peritonitis in pericyte-depleted mice. At 6 dpi, consistent with our previous experiments, all pericyte-depleted mice that were infected with wild-type *B. ovis* developed peritonitis (based on both gross and histologic findings), whereas none of the pericyte-depleted mice infected with *B. ovis* ∆*virB2* (strain lacking a functional T4SS) or inoculated with inactivated, but structurally intact, gamma-irradiated *B. ovis* developed peritonitis (Table S1).

### Modulation of inflammation by pericytes also plays a role in the outcome of infection with other bacterial pathogens

To confirm our *in vitro* observation that modulation of inflammatory response by pericytes occurs during infection with other organisms, pericyte-depleted mice and non-depleted controls were intraperitoneally infected with *L. monocytogenes*. Pericyte-depleted mice developed fibrinous peritonitis at 48 hpi, whereas none of the non-depleted developed grossly detectable changes in the peritoneal cavity when infected with *L. monocytogenes* (Fig. 5G). Pericyte-depleted mice also developed more severe clinical signs (Fig. 5H). Similar to our previous observations with *B. ovis*-infected mice, pericyte-depleted mice infected with *L. monocytogenes* also had higher levels of CCL-2 in the serum when compared with non-depleted infected mice (Fig. 5I).

**Fig 6:**
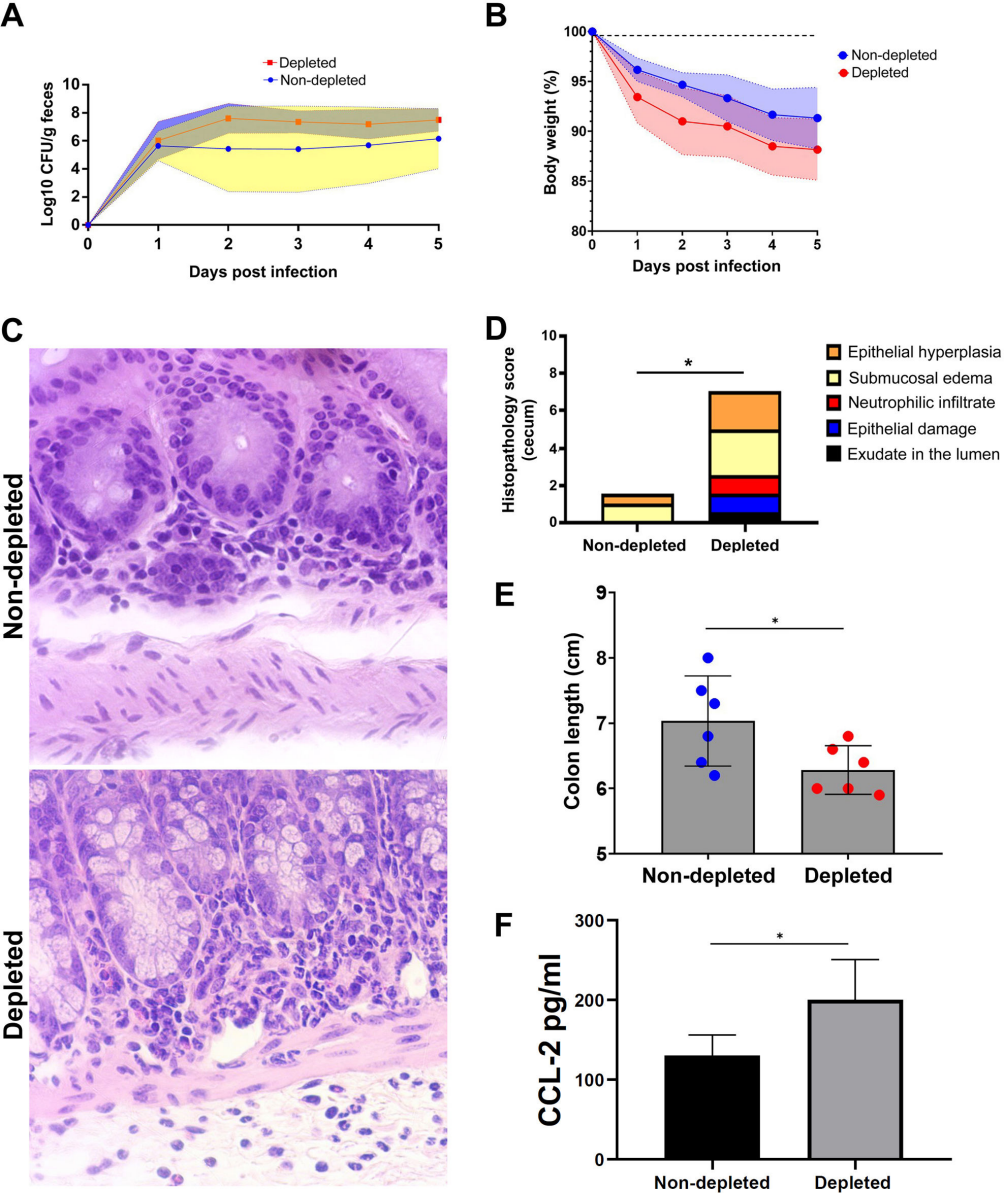
Pericytes modulate the inflammatory response to *Citrobacter rodentium in vivo*. (**A**) Shedding of *C. rodentium* in the feces of experimentally infected non-depleted (*n* = 6) and pericyte-depleted (*n* = 6) mice. (**B**) Body weight loss of non-depleted (*n* = 6) and pericyte-depleted (*n* = 6) mice infected with *C. rodentium*. (**C**) Neutrophilic infiltration in the cecal lamina propria of pericyte-depleted mice infected with *C. rodentium* and absence of inflammation in non-depleted mice infected under the same experimental conditions. (**D**) Histopathology scores of ceca from pericyte-depleted (*n* = 6) and non-depleted (*n* = 6) mice infected with *C. rodentium* at 6 dpi (*P < 0.05). (**E**) Colon length in pericyte-depleted (*n* = 6) and non-depleted (*n* = 6) mice infected with *C. rodentium* (*P < 0.05). (**F**) Serum levels of CCL-2 in non-depleted (*n* = 6) and pericyte-depleted (*n* = 6) mice infected with *C. rodentium* (*P < 0.05).

Considering that neither *B. ovis* nor *L. monocytogenes* have the mouse as their preferential host and that the intraperitoneal route is not a natural route of infection, we next investigated the role of pericyte modulation of inflammation using a mouse pathogen through a natural route of infection. *Citrobacter rodentium* is a mouse-specific pathogen that shares many similarities with enteropathogenic *E. coli* and that induces a proliferative response of the cecal and colonic epithelium, but it is not invasive and does not trigger significant intestinal inflammation (44). *C. rodentium* colonized both pericyte-depleted and non-depleted mice similarly (Fig. 6A), resulting in similar weight losses in these two groups of mice (Fig. 6B). As expected, non-depleted mice inoculated with 10^9^ CFU of *C. rodentium* did not develop significant intestinal inflammation. In contrast, pericyte-depleted mice developed a neutrophilic acute typhlitis that resulted in significantly higher histopathology scores (Fig. 6C and D) and had significantly shorter colons (Fig. 6E), a measure of colitis. Similar to other *in vivo* infections in this study, pericyte-depleted mice had higher levels of CCL-2 during the course of infection with *C. rodentium* (Fig. 6F).

Together, these results indicate that the modulation of the inflammatory response by pericytes *in vivo* is not restricted to *B. ovis*, as demonstrated by infection with *L. monocytogenes* (a Gram-positive pathogen) or *C. rodentium*, which is a mouse pathogen delivered through a natural route of infection. Furthermore, these results demonstrate that modulation of inflammation by pericytes may take place in sites other than the peritoneal cavity, as demonstrated in the intestinal mucosa of pericyte-depleted mice infected with *C. rodentium*.

### Pericyte depletion increases systemic bacterial dissemination in mice

**Fig 7:**
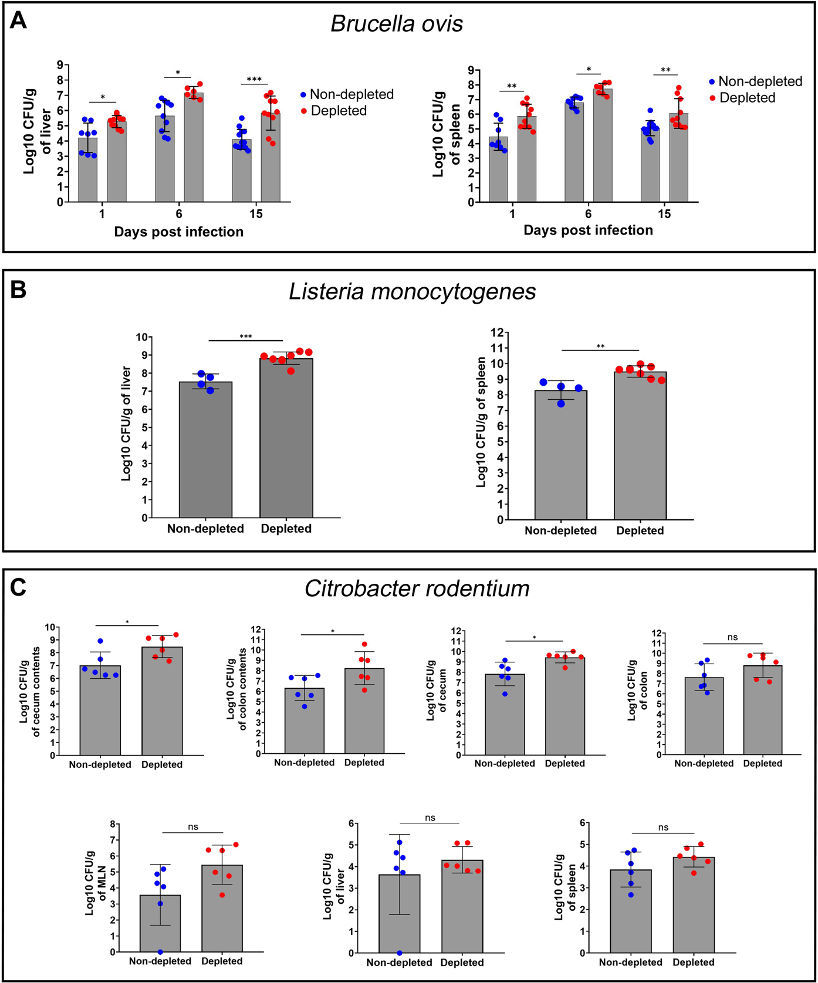
Pericyte depletion increased systemic bacterial dissemination in mice. (**A–C**) Bacterial loads in pericyte-depleted (*n* = 6–10; as indicated by red dots) or non-depleted (*n* = 4–13; as indicated by blue dots) mice experimentally infected with (**A**) *Brucella ovis* at 1, 6, and 15 dpi; (**B**) *Listeria monocytogenes* at 2 dpi; or (**C**) *Citrobacter rodentium* at 6 dpi. *P < 0.05, **P < 0.01, and ***P < 0.001; ns, non-significant (*P* > 0.05).

To determine whether pericytes limit *B. ovis* dissemination from the site of infection, we determined bacterial loads in the liver and spleen of control and pericyte-depleted mice. At 1 dpi, pericyte-depleted mice had approximately 10-fold more *B. ovis* in the liver and spleen (Fig. 7A), suggesting that pericytes contribute to controlling dissemination of bacterial infection. This difference persisted at 6 dpi and increased by 15 dpi when it reached approximately 10- or 100-fold difference in the liver or spleen, respectively. Interestingly, the attenuated *B. ovis* ∆*virB2* strain, which is deficient in its T4SS, was also recovered in approximately 10-fold higher numbers from the livers and spleens of pericyte-depleted mice compared with control mice at 6 dpi ( Fig. S3A and B). Thus, the absence of pericytes did not enable the *B. ovis* T4SS mutant to replicate at wild-type levels in tissue (Fig. S3A and B) but rather permitted it to disseminate from the inoculation site to the liver and spleen. Pericyte-depleted mice had significantly lower numbers of *B. ovis* ∆*virB2* in the liver and spleen, when compared with the wild-type strain (Fig. S3A and B), indicating that pericyte-depleted mice were still able to largely control *B. ovis* ∆*virB2*.

These results may suggest that one possible explanation for the increased inflammatory pathology in the pericyte-depleted mice is the increased bacterial infection levels in tissue. To explore this possible mechanism, C57BL/6 mice were intraperitoneally infected with 10^7^, 10^8^, or 10^9^ CFU of wild-type *B. ovis* and euthanized at 1, 3, or 7 dpi for assessment of inflammatory responses. None of the challenge doses at any of the time points elicited gross or microscopic changes in the peritoneal cavity (Fig. S3C). These results indicated that the *B. ovis*-induced peritonitis in pericyte-depleted mice is due to absence of pericytes, rather than to the higher bacterial loads. As expected, *B. ovis* CFU numbers in the spleen and liver were higher in mice subjected to higher challenge doses (Fig. S3D and E), indicating that systemic colonization in mice is dose-dependent under these experimental conditions. Importantly, at the highest *B. ovis* challenge dose in wild-type mice, which yielded similar *B. ovis* CFU numbers in tissues as pericyte-depleted mice infected with a lower dose (between 10^7^ and 10^8^ in the spleen), there were neither gross nor histological signs of peritonitis (Fig. 7A; Fig. S3D and E).

We observed a similar increase in bacterial colonization of liver and spleen after IP infection of pericyte-depleted mice with *L. monocytogenes* (Fig. 7B), compared to control mice. Further, pericyte depletion of mice increased tissue colonization after inoculation with the intestinal pathogen *C. rodentium,* resulting in increased bacterial recovery from cecum and colon, as well as from systemic sites of infection such as mesenteric lymph nodes, liver, and spleen (Fig. 7C).

Together, these results suggest that pericytes limit systemic dissemination and colonization of various bacterial pathogens, including *B. ovis*, *L. monocytogenes*, and *C. rodentium,* in experimentally infected mice. Importantly, higher levels of inflammation are not due to higher CFU numbers but actually to depletion of pericytes.

### Modulation of endothelial inflammatory response by pericytes is mediated by connexin 43

**Fig 8:**
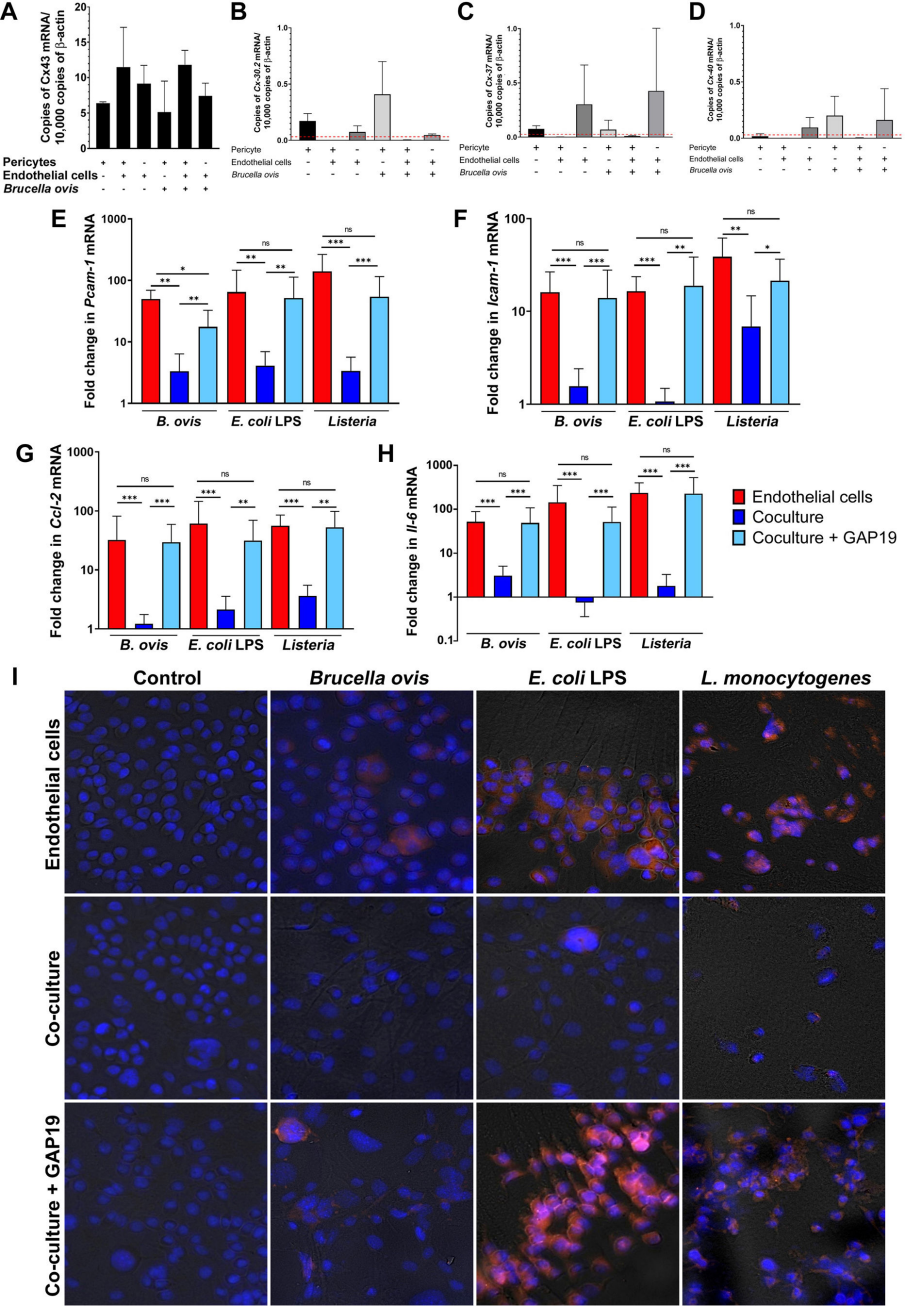
Modulation of endothelial inflammatory response by pericytes is mediated by connexin 43. (**A through D**) Profile of transcription of connexin 43 (*Cx43*), connexin 30.2 (*Cx30.2*), connexin 37 (*Cx37*), and connexin 40 (*Cx40*) by endothelial cells, pericytes, and co-culture of these two cells, either uninfected or infected with *Brucella ovis*. The red dashed lines indicate the detection limits. (**E through H**) Effect of Cx43 inhibitor (GAP19) on transcription of adhesion molecules and proinflammatory cytokines by endothelial cells co-cultured with pericytes, including (**E**) *Pcam-1*, (**F**) *Icam-1*, (**G**) *Ccl-2*, and (**H**) *Il-6*. (**I**) Effect of Cx43 inhibitor (GAP19) on expression of PECAM-1 (red) by endothelial cells co-cultured with pericytes and inoculated with *Brucella ovis*, *Escherichia coli* LPS, or *Listeria monocytogenes*. Data are representative of three independent experiments performed in triplicates.

Pericytes and endothelial cells interact with each other through peg-socket junctions (7), which are rich in gap junctions and hemichannels composed of transmembrane connexins (Cx), particularly Cx43 (8, 9, 45, 46), although there are reports that pericytes may also express Cx30.2 (47) Cx37, and Cx40 (45). Therefore, we first assessed constitutive transcription of Cx43, Cx37, Cx30.2, and Cx40 by endothelial cells, pericytes, and co-cultured endothelial cells and pericytes. Transcription of Cx43 was relatively abundant and similar among the individual cultures or co-cultured endothelial cells and pericytes, and it was not affected by inoculation with *B. ovis* (Fig. 8A). In contrast, transcripts for Cx37, Cx30.2, and Cx40 were either scarce or absent under all experimental conditions (Fig. 8B through D).

Gap19 is a peptide that blocks gap junctions (48). In order to test the hypothesis that gap junctions, particularly those formed by Cx43, are involved in the crosstalk between endothelial cells and pericytes, co-cultured pericytes and endothelial cells or endothelial cells alone were treated with Gap19 for 24 hours and then subjected to various inflammatory stimuli, including *B. ovis* (MOI 100), *L. monocytogenes* (MOI 5), and *E. coli* LPS (10 pg/mL), or sterile RPMI-control. In an initial experiment, we measured LDH release by cells treated with Gap19 and found that LDH levels in culture treated with Gap19 were less than 10% of those measured in negative control cultures (Fig. 1C), suggesting that Gap19 is not cytotoxic to the host cells. We then demonstrated that Gap19 treatment prevented the pericyte-induced downregulation of adhesion molecule expression (*Pecam-1* and *Icam-1*) in response to inflammatory stimuli, as measured by mRNA and protein expression at 24 hpi (Fig. 8E and F). A similar result was obtained when measuring transcription of pro-inflammatory genes (*Ccl-2*, *Il-6*) (Fig. 8G and H). Therefore, co-cultured endothelial cells and pericytes treated with Gap19 expressed PECAM-1 in similar levels when compared with endothelial cells alone subjected to various inflammatory stimuli: *B. ovis* (MOI 100), *L. monocytogenes* (MOI 5), or LPS (10 pg/mL) at 24 hpi. Conversely, as observed in our previous experiments, endothelial cells co-cultured with pericytes in the absence of Gap19 did not express high levels of PECAM-1 in response to these stimuli (Fig. 8I).

**Fig 9:**
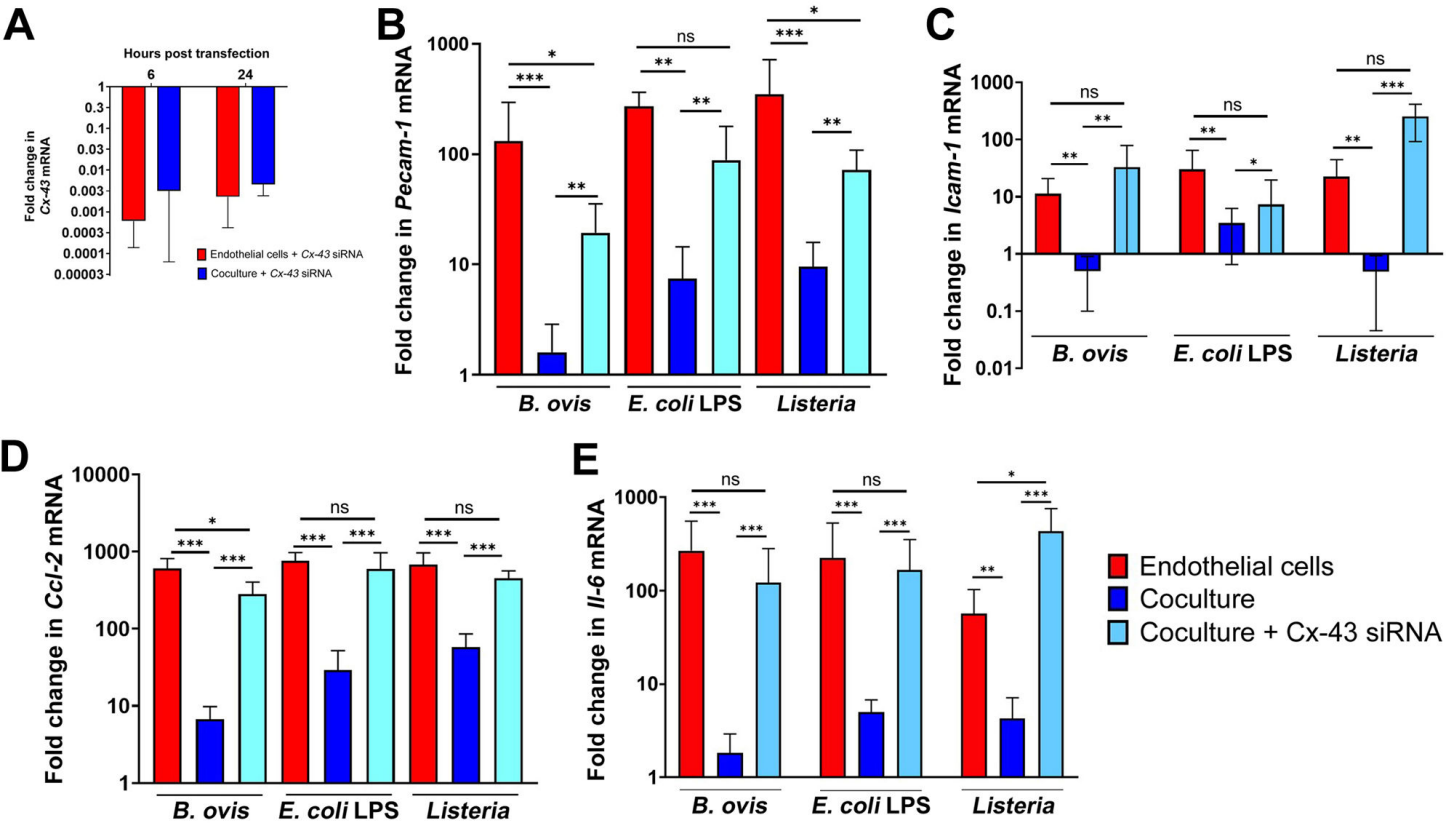
Modulation of endothelial inflammatory response by pericytes is mediated by connexin 43. (**A**) Silencing of Cx43 expression by Cx43 siRNA in endothelial cells or endothelial cells co-cultured with pericytes at 6 or 24 hours after transfection. (**B through E**) Effect of Cx43 siRNA on transcription of adhesion molecules and proinflammatory cytokines by endothelial cells co-cultured with pericytes, including (**B**) *Pecam-1*, (**C**) *Icam-1*, (**D**) *Ccl-2*, and (**E**) *Il-6*. Data are representative of three independent experiments performed in triplicate. *P < 0.05, **P < 0.01, and ***P < 0.001; ns, non-significant (*P* > 0.05).

To demonstrate that the modulation of endothelial inflammatory responses by pericytes is specifically mediated by Cx43, we used siRNA to suppress expression of *Cx43*, which resulted in a marked decrease of *Cx43* transcripts in endothelial cells and pericytes either alone or in co-culture (Fig. 9A). Then, co-cultured endothelial cells and pericytes or endothelial cells alone were subjected to pro-inflammatory stimuli: *B. ovis* (MOI 100), *L. monocytogenes* (MOI 5), *E. coli* LPS (10 pg/mL), or sterile RPMI-control. Strikingly, silencing of *Cx43* expression restored transcription levels of *Pcam-1*, *Icam-1*, *Ccl-2*, and *Il-6* by endothelial cells co-cultured with pericytes and stimulated with *B. ovis* (MOI 100), *L. monocytogenes* (MOI 5), or *E. coli* LPS (10 pg/mL) to levels similar to those of endothelial cells alone in most cases (Fig. 9B through E).

Together, these results indicate that the modulation of endothelial inflammatory responses by pericytes is mediated by Cx43.

## DISCUSSION

**Fig 10:**
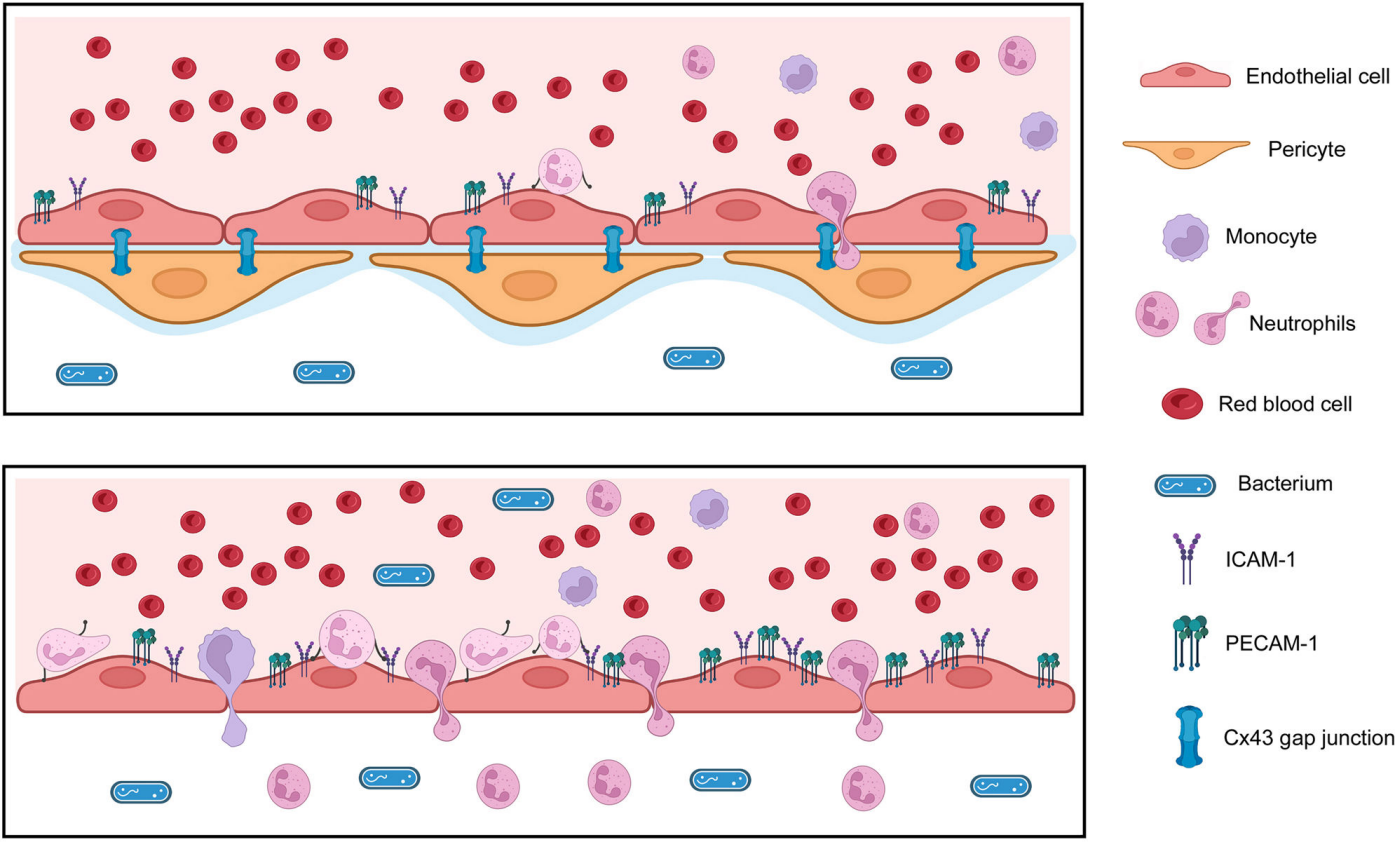
Schematic representation of the interaction of pericytes and endothelial cells through Cx43 gap junctions modulating expression of adhesion molecules and inflammation characterized by endothelial transmigration of neutrophils and monocytes, which is enhanced in the absence of pericytes.

In this study, we demonstrated a previously unknown function of pericytes modulating endothelial inflammatory responses to bacterial infections *in vitro* and *in vivo*, diminishing bacterial-elicited inflammation. In the absence of pericytes, endothelial cells displayed a much stronger pro-inflammatory response to bacterial stimuli, as demonstrated by higher expression of adhesion molecules and pro-inflammatory cytokines and chemokines. Furthermore, we demonstrated that interaction of pericytes and endothelial cells in this context is mediated by Cx43 (Fig. 10). This mechanism is likely relevant for preventing excessive tissue damage induced by inflammation, indirectly contributing to restoration of host homeostasis. Importantly, pathogenic bacteria cause damage not only through their virulence factors but also by inducing inflammatory responses. The blood vessels play a key role during the host inflammatory response by transporting leukocytes and soluble inflammatory mediators (49). In addition, endothelial cells are a source of several cytokines, chemokines, and other molecules that are required for the innate immune response (34, 50). However, a “cytokine storm” may drastically impact morbidity and mortality due to severe clinical manifestations, for example, in cases of sepsis (49). This study demonstrated that pericytes may be essential to prevent exacerbated and potentially deleterious endothelial cell-mediated pro-inflammatory mechanisms.

We initially demonstrated pericyte modulation of endothelial inflammatory response against *B. ovis* infection. *Brucella* spp. are considered stealthy pathogens that usually do not trigger a detectable inflammatory response during the acute phase of infection (51). Thus, *B. ovis* was elected as a model organism to assess the role of pericytes in inflammation due to its low intrinsic pro-inflammatory potential. Although *Brucella abortus* invades and survives within cultured endothelial cells infected *in vitro* (34), there are no previous studies on the interaction of *Brucella* spp. and pericytes. However, the studies presented here may not accurately reflect the role that pericytes play during infections with *Brucella* spp. other than *B. ovis*, including *B. melitensis*, *B. abortus*, or *B. suis*. Furthermore, in this study, we also employed *L. monocytogenes*, a Gram-positive facultative intracellular bacterial pathogen (52), and purified *E. coli* LPS, which is a strong TLR4 agonist (25). Therefore, we demonstrated that this mechanism was not restricted to one specific bacterial agent since stimulation with *B. ovis*, *L. monocytogenes*, and *E. coli* LPS induced upregulation of adhesion molecules (ICAM-1 and PECAM-1) and pro-inflammatory genes (*Il-6*, *Ccl-2*, and *Tnf-α*) in endothelial cells, but when endothelial cells were co-cultured with pericytes, these responses were markedly diminished. Endothelial cells are involved in several physiologic functions and pathologic responses, including metabolic homeostasis, vascular hemodynamics, vascular permeability, coagulation, and cell extravasation (leukocyte trafficking) (1). Inflammatory mechanisms specifically performed by endothelial cells are critical in many conditions such as septic shock (53) and thrombosis (54). Inflammatory signals from exogenous or endogenous sources may stimulate endothelial cells to escalate inflammation through the production of cytokines/chemokines (such as IL-6, MCP1, and TNF-α) and adhesion molecules (ICAM-1, VCAM-1, PECAM-1, and E-selectin) (55). Our *in vitro* results are in good agreement with a previous study, which demonstrated that pericyte loss in the retina increases leukocyte infiltration and upregulates expression of inflammatory genes and adhesion molecules by retinal endothelial cells (19).

Intraperitoneal infection of pericyte-depleted mice with wild-type *B. ovis*, a stealthy pathogen, resulted in the development of clinical signs, fibrous peritonitis, and mortality. These results unequivocally demonstrate a relevant role of pericytes in regulating inflammation *in vivo*. Clinical signs and mortality did not occur in wild-type mice experimentally infected with high doses of *B. ovis* (10^7^, 10^8^, or 10^9^ CFU/animal), which was expected in *Brucella* spp.-infected mice (51). Importantly, even after these very high challenge doses, *B. ovis* was not capable of inducing peritonitis in wild-type mice with an intact population of pericytes. Furthermore, although high challenge doses of wild-type *B. abortus* or *B. microti*, a highly pathogenic species to mice, may result in lethal infections (56, 57), even under those conditions, there are no previous reports of any gross or histological changes in the peritoneal cavity or peritonitis. Therefore, the interpretation of our results points to the fact that a reduction in the pericyte population rendered mice more sensitive to *B. ovis-*elicited inflammation. However, this is not a species-specific phenomenon since another intracellular bacterial pathogen, the Gram-positive *L. monocytogenes*, which usually does not trigger a detectable inflammatory response in the abdominal cavity, induced peritonitis in pericyte-depleted mice. Thus, the modulation of endothelial cell inflammatory responses demonstrated in our initial experiments in cultured cells correlated very well with the outcome of infection *in vivo* and *in vitro*. Conversely, a pro-inflammatory response obviously plays a positive role for controlling pathogens, such as *L. monocytogenes* (58), but in spite of a stronger local inflammatory response, there were higher bacterial loads at systemic sites of infection, indicating that the absence of pericytes results in more systemic dissemination of these pathogens, which is somewhat expected since pericytes directly maintain vascular integrity (59). Limitations of *B. ovis* and *L. monocytogenes* models of infection in the mouse include the fact that these pathogens do not have the mouse as a preferred host and that the inoculation route (intraperitoneal) is not a natural route of infection. Therefore, we performed additional *in vivo* experiments with *C. rodentium*, a mouse-specific pathogen (44), which was experimentally inoculated through a natural route of infection (gastrointestinal tract). Interestingly, although *C. rodentium* does not cause significant inflammation in non-depleted mouse, as extensively demonstrated (44), it elicits an acute intestinal inflammatory reaction with edema and neutrophilic infiltration in pericyte-depleted mice, demonstrating that modulation of inflammation by pericytes occurs in various anatomic compartments upon stimulation with different bacterial agents.

In this study, the absence of pericytes increased expression of adhesion molecules (ICAM-1 and PECAM-1) by endothelial cells in response to bacterial stimuli. During the inflammatory process, leukocytes cross the endothelial layer by anchoring themselves through adhesion molecules such as PECAM-1 and ICAM-1 (60). *In vivo* infection of pericyte-depleted mice with wild-type *B. ovis* by the intraperitoneal route resulted in fibrinous peritonitis and infiltration of macrophages and neutrophils. Importantly, as a stealthy pathogen, *B. ovis* does not elicit a detectable inflammatory response in the abdominal cavity when inoculated intraperitoneally (51). Recruitment of leukocytes from the bloodstream and the sequence of adhesive contacts of these cells with endothelial cells that will ultimately allow leukocytes to migrate to the site of injury are essential steps for host defense. This innate immune reaction must be well orchestrated to avoid migration of excessive numbers of inflammatory cells and consequently tissue damage (60). In extreme cases, an overwhelming pro-inflammatory response may result in devastating consequences such as in sepsis (61). Our findings are also in good agreement with previous studies (19) that indicated that pericyte depletion may favor macrophages and neutrophil infiltration in tissues. Therefore, increased expression of adhesion molecules by endothelial cells in the absence of pericytes likely had a relevant role for the development of peritonitis in our *in vivo* model of infection.

In addition to their structural and the physiological functions, pericytes have been implicated in disease development and recrudescence, including neurological disorders, cancer, and diabetic-related conditions (13–15). There are also evidences that pericytes may be implicated in human cytomegalovirus infection (62, 63). Although under specific experimental conditions, pericytes may respond to bacterial stimuli by producing proinflammatory chemokines and cytokines (64, 65), this study demonstrated that pericytes are not preferential target cells for *B. ovis* infection. Our experimental evidence indicates that pericytes are rarely infected and are not permissive to either *B. ovis* or *L. monocytogenes* intracellular growth. In contrast, pericytes are susceptible to infection by *Bartonella henselae*. Cultured human pericytes are permissive to invasion by *B. henselae*, which induces pathological angiogenesis resulting in a condition known as angiomatosis. *B. henselae*-infected pericytes produce higher levels of vascular endothelial growth factor, which may be responsible for the abnormal angiogenesis induced by this pathogen (66). In contrast, *B. ovis* can invade and replicate in endothelial cells, which is in agreement with a previous study that demonstrated infection of human endothelial cells with *B. abortus* (34). Therefore, the modulation of endothelial cell inflammatory responses by pericytes is likely to be a steady-state mechanism that prevents unwanted or exacerbated inflammation driven by endothelial cells. In other words, under these conditions, pericytes do not necessarily need to sense invading bacteria to exert their modulatory function. Importantly, we demonstrated that Cx43, presumably in gap junctions, is responsible for the crosstalk between pericytes and endothelial cells, which is compatible with a steady-state modulation by pericytes. During inflammation, the crosstalk between endothelial cells and pericytes maintains the inflammatory process tightly regulated. Indeed, disturbances of this mechanism of cell-to-cell communication may cause microvascular dysfunction, such as micro-hemorrhages (19). For instance, decreased pericyte coverage over blood vessels of the subcortical white matter of Alzheimer’s disease patients was related to changes in vascular density and high accumulation of blood-derived extravascular fibrin deposits (67). Interestingly, HIV-1 infection and latency in pericytes impair the host DNA damage response favoring chronic neuroinflammatory conditions (68). Furthermore, expression of adhesion molecules by endothelial cells is critical for recruitment, activation, and influx of leukocytes in the site of inflammation, representing a key step for an effective innate immune response (35, 69). Importantly, endothelial cells are capable of triggering a pro-inflammatory response to stimulation with PAMPs (34, 50) since endothelial cells are known to express TLR-4 and TLR-2 (70). In this study, bacterial infection in the context of pericyte depletion triggered a severe inflammatory process that resulted in fibrin deposition and intense inflammatory cell infiltration at the site of infection.

In conclusion, this study demonstrated that pericytes play a role controlling endothelium-mediated inflammatory mechanisms both *in vivo* and *in vitro* in response to bacterial stimuli. Our results also support the notion that the modulation of endothelial cell inflammatory response by pericytes is mediated by Cx43. Therefore, we hypothesize that pericytes promotes a steady-state modulation of endothelial cells’ inflammatory threshold, which may be critical for preventing exacerbated innate immune reactions that may cause tissue damage resulting from an overwhelming production of proinflammatory cytokines and chemokines and increased infiltration and activation of leukocytes.

## MATERIALS AND METHODS

### Bacterial strains and culture conditions

Wild-type *B. ovis* ATCC 25840, *B. ovis* ∆*virB2* (71), *B. ovis* expressing *mCherry* fluorescent protein (*B. ovis* mCherry), wild-type *L. monocytogenes* 10,403s kindly provided by Dr. Daniel A Portnoy (UC Berkeley), and *Citrobacter rodentium* ATCCBAA-2623 were used in this study. *B. ovis* strains were grown on tryptose soy agar supplemented with 1% hemoglobin (hTSA) (Becton Dickinson, Brazil) in a humidified incubator at 37°C with 5% CO_2_ for 3 days. *L. monocytogenes* was grown on brain heart broth (BHI) agar plates or broth. *C. rodentium* was grown on MacConkey agar or Luria-Bertani (LB) broth. Inocula were prepared by suspending the colonies harvested from plates in sterile PBS (phosphate buffer saline, pH 7.4, Sigma-Aldrich) or BHI broth. The quantity of CFU/mL of inoculum was estimated by spectrophotometry at 600 nm (SmartSpec Plus Bio-Rad, USA) and confirmed by counting individual colonies grown on plate after incubation of 10-fold serial dilutions.

Bacterial suspensions of inactivated *B. ovis* ATCC 25840 were prepared by irradiating a PBS suspension containing 10^9^ CFU/mL of bacteria with 15 Kgray of gamma radiation for 12 hours (Laboratory of Gamma Irradiation at the Center for Development of Nuclear Technology—CDTN-UFMG). Bacterial inactivity was confirmed by plating aliquots of gamma-irradiated bacteria onto hTSA.

### Genetic background of mice and genotyping

The NG2creER, iDTR (inducible DTR), and Nes-GFP (Nestin expressing GFP) C57BL/6 transgenic mice were kindly provided by Dr. A. Birbrair from the Institute of Biological Science (ICB) at UFMG or purchased from Jackson Laboratory. Wild-type C57BL/6J mice were purchased from Jackson Laboratory. By crossbreeding homozygous iDTR and heterozygous NG2creER C57BL/6, the double transgenic lineage NG2creER × iDTR was obtained, whereas the CRE-negative C57BL/6 mice (iDTR mice) were used as negative controls. Mice were maintained at cages under controlled temperature and humidity (25°C, 70%) and had free access to filtered water and commercial feed.

Mice were genotyped by PCR using primers targeting the CRE gene (5′-AACATGCTTCATCGTCGG-3′ and 5′-TTCCGATCATCAGCTACACC-3′) as described (72). PCR mixture contained 15.0 µL of PCR Supermix (Thermo Fisher Scientific, USA), 1.0 µM of each primer, 4.0 µL of DNA, and supplementation with 1.0 U of Taq DNA polymerase recombinant (Thermo Fisher Scientific, USA). PCR reaction was set up as follows: 94°C for a 5-min, 35 cycles at 94°C for a 30-s, 55°C for a 30-s, 72°C for a 1-min, and a 7-min final elongation step at 72°C. A 412-bp amplicon was expected from positive DNA on a 1.5% agarose gel.

### Cell lines, culture, and co-culture conditions

Mouse brain vascular pericyte primary cells (iXCells), mouse endothelial cell line EOMA (ATCC), and mouse macrophage cell line J774 (ATCC) were maintained in RPMI supplemented with 10% fetal bovine serum (FBS) or pericyte media (iXCells) supplemented with 10% FBS and incubated at 37°C with 5% CO_2_. For co-culture experiments, endothelial cells and pericytes were co-cultured in the proportion of 2:1 (endothelial cells:pericytes) for 24 hours prior to the experimental treatments.

Cells were seeded (10^5^ cells/well) in 96-well plates 2 hours prior to infection with *B. ovis* at a multiplicity of infection (MOI) of 100 or 1,000. For co-cultures, endothelial cells and pericytes were seeded at 10^5^ cells/well and 5 × 10^4^ cells/well, respectively, in a 24-well plate, and inoculated with *B. ovis* (MOI 100) and *L. monocytogenes* (MOI 5) or stimulated with 10 pg/mL of purified *E. coli* LPS (eBioscience). Soon after inoculation plates were centrifuged (170 × *g*) at room temperature, followed by incubation at 37°C with 5% CO_2_ for 30 min. The cells were then washed twice with PBS and then further incubated with RPMI supplemented with 0.1 mg/mL of gentamycin. Sterile RPMI was used as negative control. Endothelial cells and co-cultures were treated with the gap junction blocker Gap19 (Tocris), 100 µM in each well, or sterile RPMI as negative control.

### NG2 cell depletion and mouse infection

NG2 cell depletion was performed as described (72). Briefly, 4- to 6-week-old mice received 1 mg of tamoxifen (Sigma-Aldrich) IP twice a day (12-hour intervals) for five consecutive days. Two days later (7th day of the protocol), DT, which triggers NG2cre cell depletion, was intraperitoneally injected at a single dose of 100 µg/kg of body weight. Seven days later (14th day of the protocol), mice were 6 to 8 weeks old and used for experimental infections. NG2 + pericyte-depleted C57BL/6 mice (*n* = 10) and iDTR C57BL/6 mice (*n* = 14) were IP injected with 100 µL of a suspension containing 10^7^ CFU/mL of *B. ovis* or 10^4^ CFU/mice of *L. monocytogenes* or intragastrically infected with 100 µL of a suspension containing 5 × 10^9^ CFU/mL of *C. rodentium*. Mice were subjected to euthanasia at 1, 3, and 15 dpi when infected with *B. ovis* or 2 and 6 dpi when infected with *L. monocytogenes* or *C. rodentium*, respectively. The liver and spleen were sampled for bacteriology and histopathology.

Six- to 8-week-old *Nes-*GFP C57BL/6 mice (*n* = 7) were inoculated IP with 100 µL of a suspension containing 10^7^ CFU/mL of *B. ovis mCherry*. At 24 hpi (*n* = 3) and 3 dpi (*n* = 4), mice were euthanized and sampled as described above.

For experiments with higher infectious doses, C57BL/6 were inoculated with 100 µL of a suspension containing 10^8^, 10^9^, or 10^10^ CFU/mL of wild-type *B. ovis* and subjected to euthanasia and sampling at 1, 3, and 7 dpi.

### Bacteriologic cultures

Tissue samples were placed in sterile PBS (pH 7.4) and maintained on ice until they were macerated and serially diluted (10-fold dilutions) in PBS. One hundred microliters of each dilution was plated on hTSA for *B. ovis*, LB for *L. monocytogenes*, or MacConkey agar for *C. rodentium*, in duplicates. Plates were incubated at 37°C in a humidified incubator supplemented with 5% CO_2_ for 1 to 5 days, when colonies were counted.

### RNA isolation and RT-qPCR

RNA was extracted from cell cultures (single cell lines or total RNA from cocultured cell lines) or tissue samples by TRIzol (Thermo Fisher) following the manufacturer’s instructions. One microgram of total RNA was used for cDNA synthesis using a RT Master Mix, and qPCR was performed using a Fast SYBR-Green Master Mix (Applied Biosystems) detected by the StepOnePlus real-time PCR system (Applied Biosystems). Primer sequences used in this study are described in Table S2. After 40 cycles, the Ct values were determined and normalized based on β-actin mRNA. Fold changes in expression between control and stimulated groups were determined by the ΔΔCt method (73).

### Cytotoxicity assay

LDH release was measured in cell culture supernatants using the Cytotox96 non-radioactive cytotoxicity assay (Promega), as previously described (57). Cell death was estimated as the percentage of LDH release, which was calculated using the following formula proposed by the manufacturer: percentage of LDH release = 100 × (test LDH release – spontaneous release)/(maximum release − spontaneous release).

### siRNA

Endothelial cells co-cultured with pericytes were transfected with mouse Cx43-specific and unspecific control siRNA (Santa Cruz, USA) using Xfect (Clontech, USA) according to the manufacturer’s instructions. For transfections, cells were plated at a density of 10^5^ endothelial cells and 5 × 10^4^ pericytes/well in 24-well plates 1 day prior to transfection. After transfection, cells were stimulated with *B. ovis*, *L. monocytogenes*, or *E. coli* LPS for 24 hpi as described above.

### *In situ* hybridization

Sections of the liver were processed for *in situ* hybridization using a probe targeting the mouse *Pecam-1* mRNA (Invitrogen, USA) and a commercially available kit (RNAview; Invitrogen, USA) according to the manufacturer’s instructions. Negative controls included a non-specific probe and replacing the probe with hybridization buffer.

### Histopathology

The liver and spleen were sampled, fixed by immersion in 10% buffered formalin for 24 hours, and embedded in paraffin. Tissue sections (3–4 μm thick) were stained with hematoxylin and eosin. Lesions in the liver and spleen (inflammation and necrosis) were scored as 0 to 3, being 0 = absent, 1 = mild, 2 = moderate, and 3 = severe, with a total score ranging from 0 to 6. In the liver, perihepatitis, thrombosis, and hepatocyte degeneration were scored as 0 or 1, being 0 = absent and 1 = present. Lesions in the cecum, including submucosal edema, necrosis, epithelial damage, and exudate, were scored 0–3 (0 = absent, 1 = mild, 2 = moderate, and 3 = severe), and the number of neutrophils was scored from 0 to 3 (0 = 0–5 neutrophils per 10 fields; 1 = 6–20; 2 = 21–50; and 3 = 51–100), with a total score ranging from 0 to 15.

### Immunohistochemistry and immunofluorescence

Tissue sections (3–4-µm thick) were deparaffinized in xylene and hydrated in decreasing ethanol concentrations. Only NG2 epitopes needed antigen retrieval, which was performed by heating the tissue fragments immersed in high pH solution (EnVision FLEX—Dako, USA) for 10 min in a pressure cooker. Tissue sections were incubated with 3% hydrogen peroxide for 1 hour and treated with 3% skimmed milk for 1 hour. A primary anti-*Brucella* rabbit polyclonal antibody (1:1,000 dilution) was incubated with the tissue sections at room temperature for 1 hour, whereas a primary anti-NG2 rabbit polyclonal antibody (Chemicon—AB5320, Sigma-Aldrich, USA) was incubated (1:250 dilution) with the tissue sections at 6°C overnight. Samples were then incubated with a secondary antibody (EnVision FLEX—Dako, USA) for 1 hour at room temperature. The chromogen was 3,3′-diaminobenzidine tetrahydrochloride (DAB) used according to the manufacturer’s instructions (EnVision FLEX—Dako, USA). Tissue sections were counterstained with Harris hematoxylin, dehydrated, and assembled for analysis.

Livers and spleens of infected of *B. ovis mCherry-*of *Nestin-*GFP mice were fixed by IV perfusion with 4% paraformaldehyde and post-fixed for 24 hours in 4% paraformaldehyde, incubated in 40% sucrose solution for 48 hours, embedded in OCT (Sigma), and stored at −20°C. Frozen 5- to 6-µm-thick tissue sections were obtained using a cryostat (Easy Path). Then, cell nuclei were stained with DAPI (Sigma). Images were acquired using an optical microscope (Leica DM3000, USA).

Cultured cells were fixed with 4% of paraformaldehyde for 10 min and incubated with the primary monoclonal anti-PECAM-1 (Santa Cruz, 1:500 dilution) for 1 hour at room temperature protected from light. The secondary antibody IgG anti-mouse with Alexa fluor 560 (Thermo Fisher, 1:400) was incubated for 2 hours at room temperature; after washing, DAPI (Sigma, 1:1,000) was incubated for 30 min at room temperature.

### Statistical analyses

Statistical analyses were performed using GraphPad Prism for Windows, version 8.4.2. Data were submitted to the Anderson-Darling test of normality. Prior to statistical analysis, bacterial counts were logarithmically transformed and compared by ANOVA followed by Tukey’s post hoc test. The averaged log numbers were used for index calculation as follows: the mean log number from treated animals (pericyte-depleted mice) was subtracted from the mean log number from untreated animals (control mice) for each analyzed organ.

Histopathological scores were analyzed as non-parametric data by means of a Mann-Whitney test, while quantifications on NG2^+^ cells were analyzed by means of Kruskal-Wallis tests followed by Dunn’s post hoc test. The frequency of peritonitis among experimental groups was analyzed by Fisher’s exact test.

## SUPPLEMENTAL MATERIAL

Supplemental materials mbio.03252-23-s0001.doc Figures S1 and S2 and Tables S1 and S2.

ASM does not own the copyrights to Supplemental Material that may be linked to, or accessed through, an article. The authors have granted ASM a non-exclusive, world-wide license to publish the Supplemental Material files. Please contact the corresponding author directly for reuse.
