# Supplementary material for: Pericytes modulate endothelial inflammatory response during bacterial infection
Source: mBio. 2024 Jan 30;15(3):e03252-23. doi: 10.1128/mbio.03252-23 (PMC10936204; doi:10.1128/mbio.03252-23)

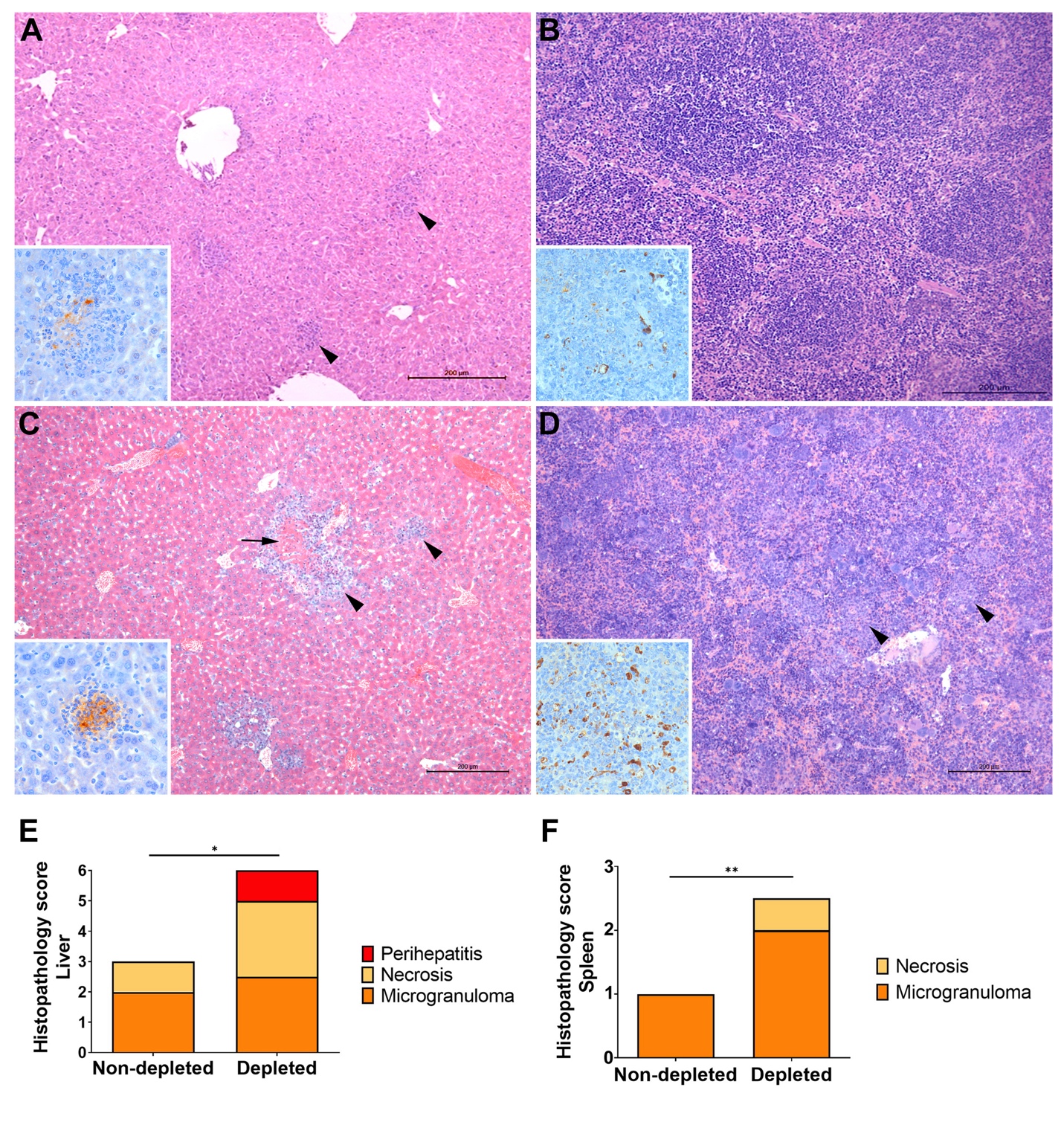


**Supplementary Figure S1.** Microscopic lesions in pericyte-depleted and non-depleted mice infected with *Brucella ovis*. (A) microgranulomas (arrowheads) in the liver of a non-depleted mouse; inset: immunolabeling of intralesional *B. ovis*. (B) spleen of a non-depleted mouse with no significant microscopic changes; inset: immunolabeling of *B. ovis*. (C) microgranulomas (arrowheads) and necrosis (arrow) in the liver of a pericyte-depleted mouse; inset: immunolabeling of intralesional *B. ovis*. (D) microgranulomas (arrowheads) in the spleen of a pericyte-depleted mouse; inset: immunolabeling of intralesional *B. ovis*. (A-D) hematoxlin and eosin stain and anti-*Brucella* sp. immunohistochemistry (insets); bars = 200 μm. (E-F) Histopathology scores for (E) the liver and (F) spleen of pericyte-depleted (n = 10) and non-depleted (n = 13) mice infected with *B. ovis*. *p < 0.05; **p < 0.01.


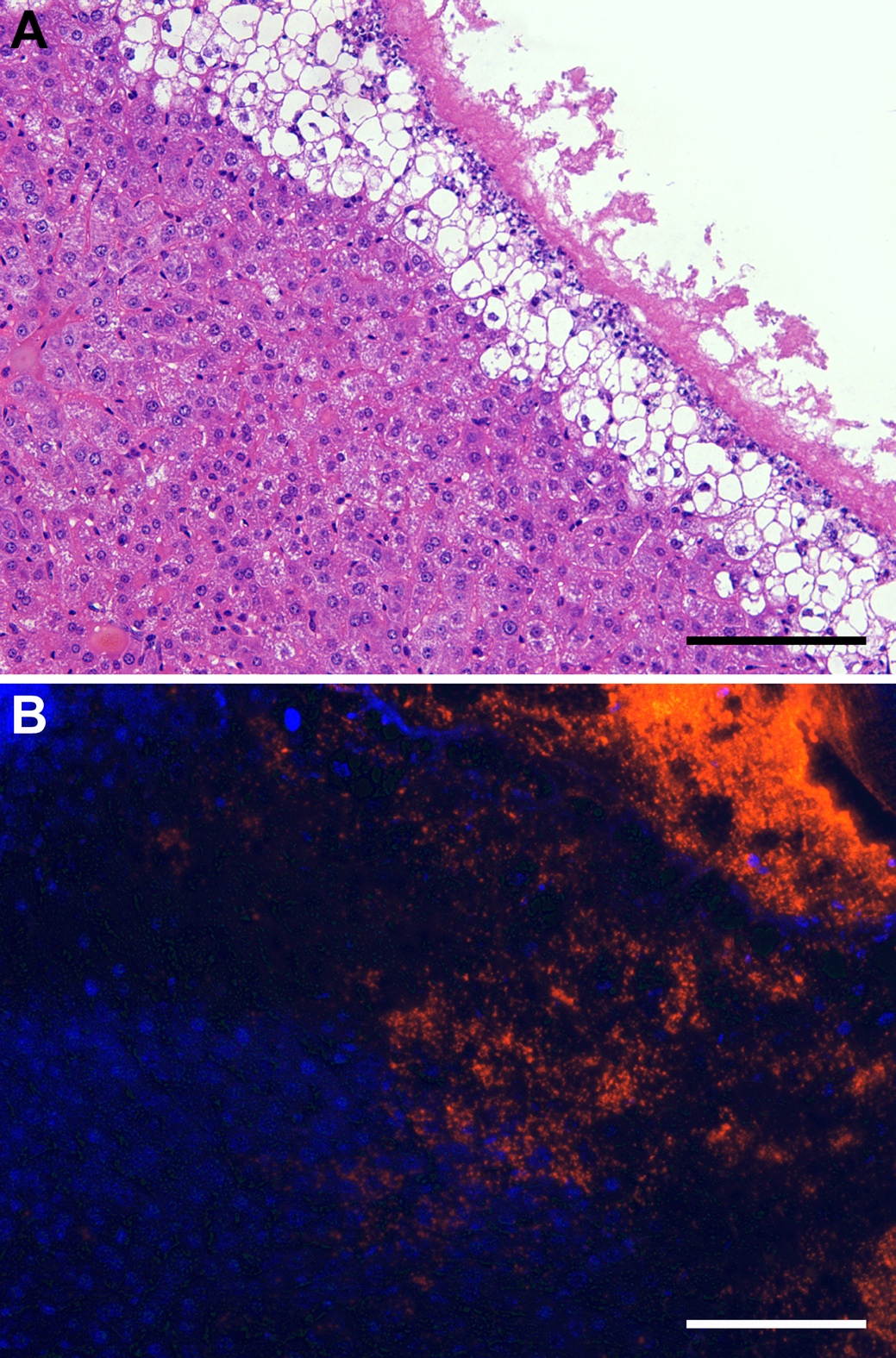


**Supplementary Figure S2.** Inflammatory and degenerative changes in the surface of the liver of pericyte-depleted mice with peritonitis associated with *Brucella ovis* infection. (A) Marked vacuolation of subcapsular hepatocytes morphologically compatible with degeneration, and accumulation of fibrin and inflammatory infiltrate on the surface of the liver. Hematoxylin and eosin, bar = 60 μm. (B) Detection of *Pecam-1* mRNA by *in situ* hybridization predominantly in the subcapsular region of the liver of pericyte-depleted mice. Bar = 60 μm.


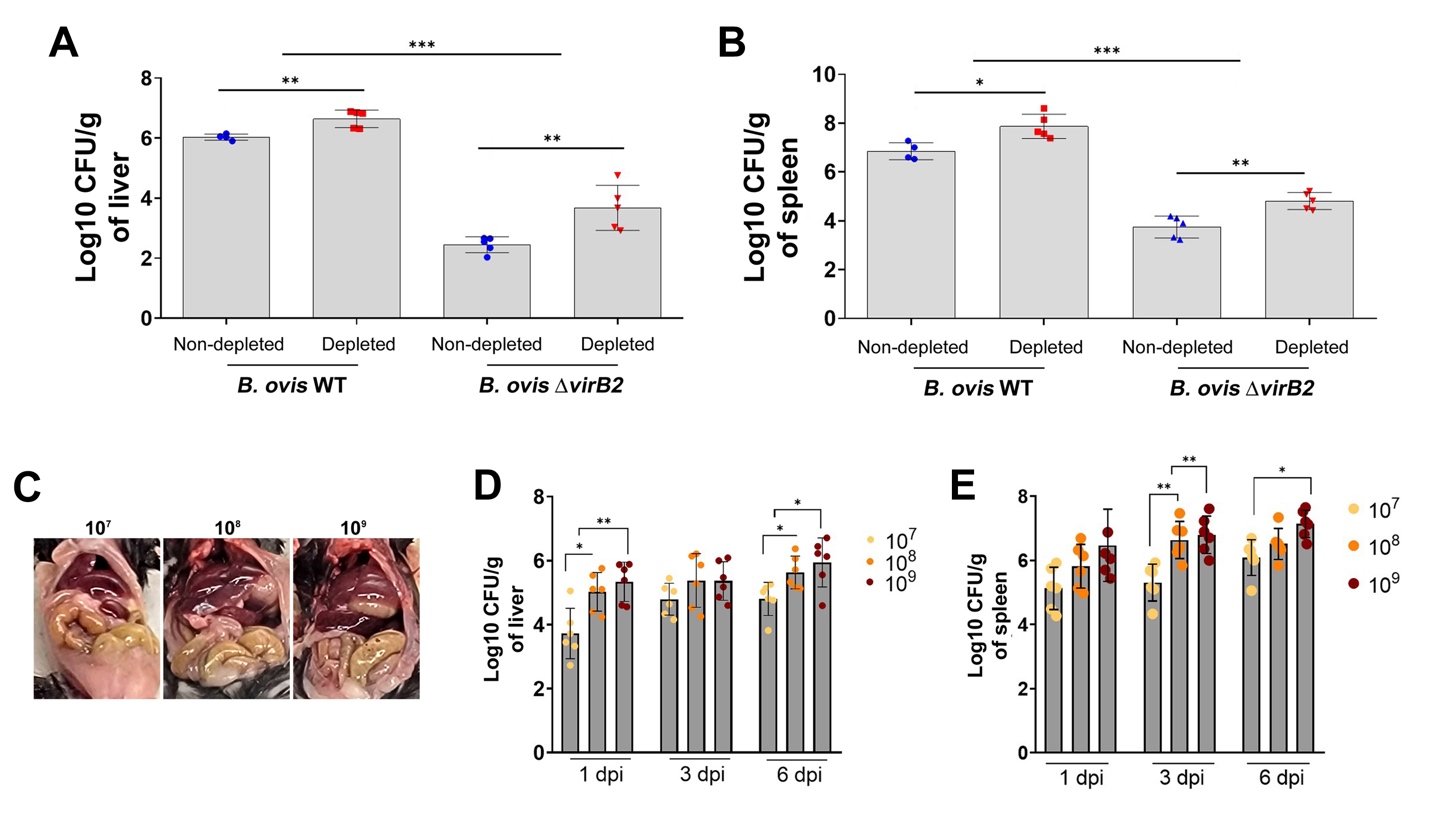


**Supplementary Figure S3.** (A, B) Bacterial loads in (A) the liver and (B) spleen of non-depleted (n =4-5) and pericyte-depleted (n =5) mice infected with wild type *Brucella ovis* or a *B. ovis* Δ*virB2* strain. (C) Peritoneal cavity of wild type C57BL/6 mice intraperitoneally infected with high doses (10^7^, 10^8^, or 10^9^) of *B. ovis*, with no gross lesions. (D-E) Bacterial loads in (D) the liver and (E) spleen of non-depleted (n =6) and pericyte-depleted (n =6) mice infected with high doses (10^7^, 10^8^, or 10^9^) of *B. ovis*. *p < 0.05; **p < 0.01; ***p < 0.001.


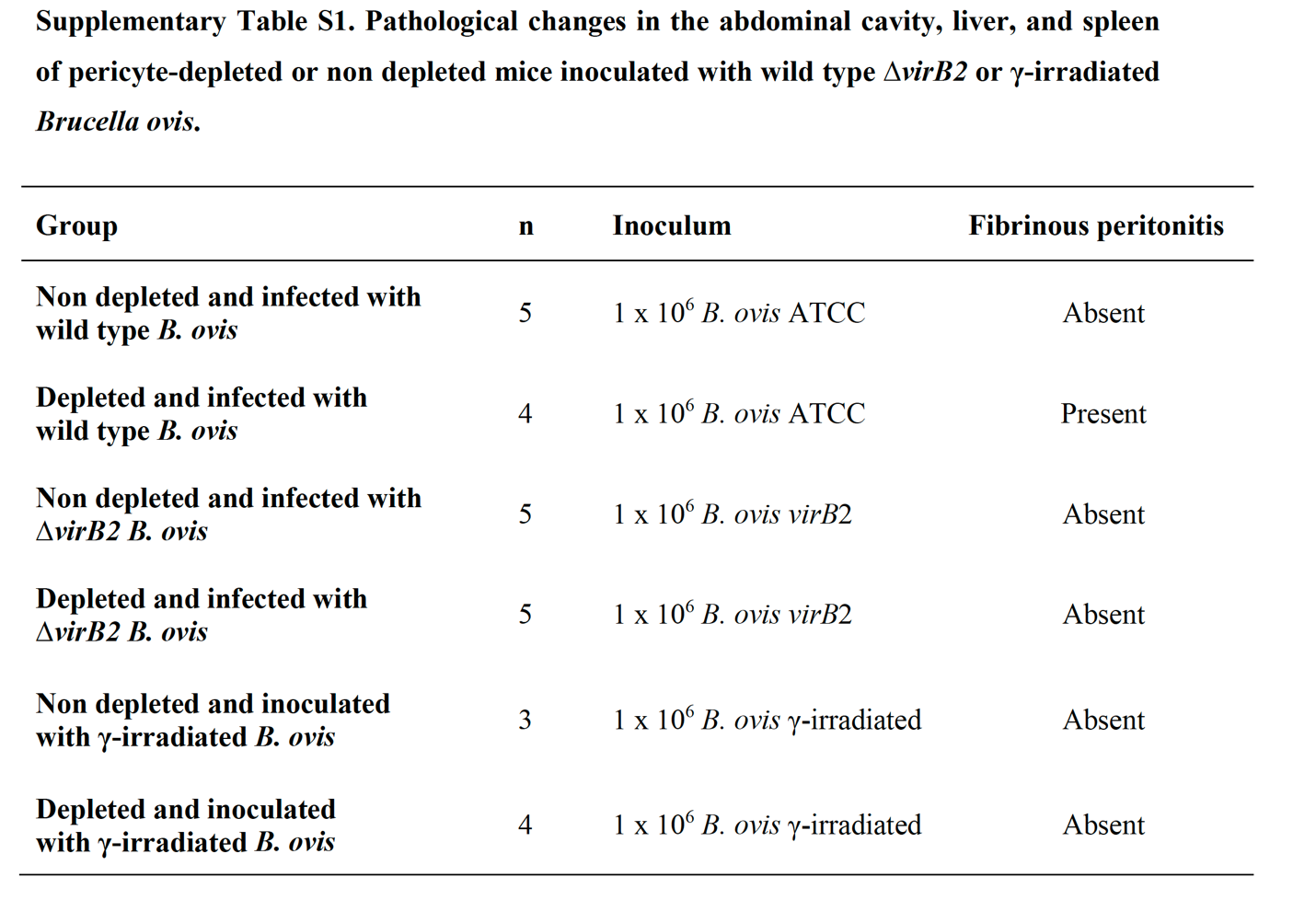


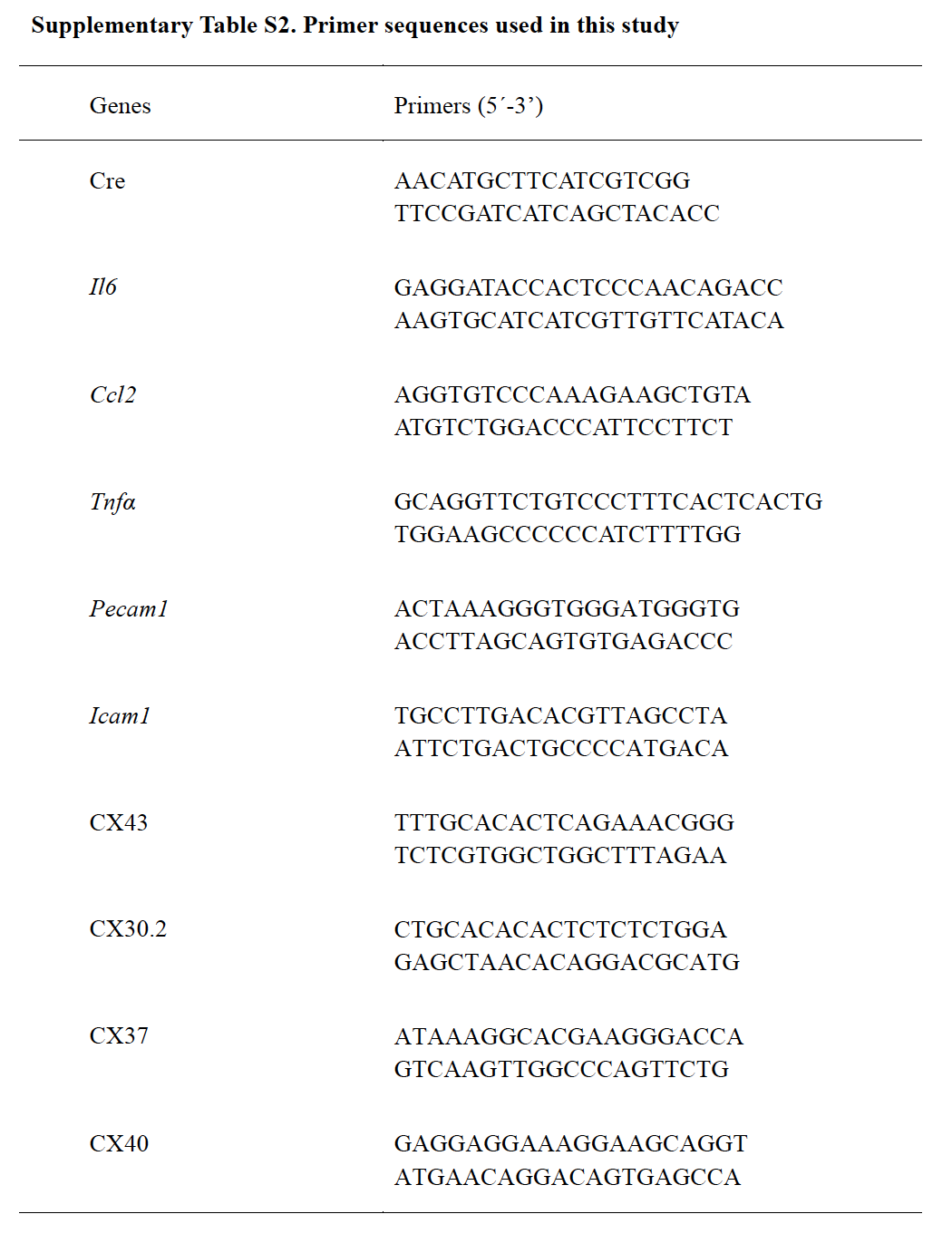

Supplement: Supplemental materials — Figures S1 and S2 and Tables S1 and S2. [file mbio.03252-23-s0001.doc]
